# Humic Acid Induces an Adaptive Stress Response During Early Signaling in Rice

**DOI:** 10.1111/ppl.70877

**Published:** 2026-04-19

**Authors:** José Nivaldo de Oliveira Sátiro, Andrés Calderín García, Andressa Fabiane Faria de Souza, Clenya Carla Leandro de Oliveira, André Luís da Silva Parente Nogueira, Rômulo Vallim dos Santos, Inês Ariane de Paiva Câncio, Orlando Carlos Huertas Tavares, Ricardo Luiz Louro Berbara, Manlio Silvestre Fernandes, Leandro Azevedo Santos

**Affiliations:** ^1^ Plant Nutrition Laboratory, Department of Soils Federal Rural University of Rio de Janeiro (UFRRJ) Seropédica Rio de Janeiro Brazil; ^2^ Soil Biological Chemistry Laboratory, Department of Soils Federal Rural University of Rio de Janeiro (UFRRJ) Seropédica Rio de Janeiro Brazil; ^3^ NIBBA—Biotechnology Innovation Hub for Agricultural Bioinputs Federal Rural University of Rio de Janeiro Seropédica Rio de Janeiro Brazil

**Keywords:** eustress, humic acids, jasmonate pathway, redox signaling

## Abstract

Humic substances (HS) are widely recognized as plant biostimulants, yet the molecular mechanisms underlying their mode of action remain incompletely defined. Here, we used RNA sequencing to investigate the early transcriptional responses of rice roots exposed for only 4 h to vermicompost‐derived humic acid (HA). Our results reveal a rapid and pronounced transcriptional reprogramming consistent with the establishment of a eustress‐like physiological state. HA treatment induced 231 genes, whereas only seven were repressed, indicating a predominantly stimulatory effect on gene expression. The induced genes were significantly enriched in functional categories related to redox homeostasis, glutathione metabolism, oxidoreductase and peroxidase activities, and cellular detoxification, suggesting an immediate adjustment of intracellular redox balance and antioxidant capacity. Concomitantly, pathways associated with phenylpropanoid metabolism, oxylipin biosynthesis, and jasmonate‐mediated signaling were activated, together with the induction of transcription factors from the WRKY and C2H2 zinc finger families, supporting early regulatory control of defense‐related networks. The coordinated activation of redox‐ and hormone‐associated pathways indicates that HA rapidly modulates ROS‐dependent signaling and integrates it with jasmonate‐centered responses. Importantly, this transcriptional signature is consistent with a eustress‐like state in which defense and adaptive mechanisms are mobilized without evidence of acute stress injury. Collectively, our findings establish a mechanistic framework for HS action in which HA functions as a chemical eustressor that rapidly reconfigures redox–hormone crosstalk, positioning reactive oxygen species signaling as a central integrative hub underlying the biostimulant and adaptive effects of humic substances in plants.

## 1 Introduction

Understanding the molecular mechanisms underlying the mode of action of humic substances (HS) in plants has challenged the scientific community for more than a century. In the early 2000s, the prevailing hypothesis suggested that humic acids (HA) derived from vermicompost contain structural fragments capable of mimicking auxins and interacting with their receptors, thereby promoting root growth (Canellas et al. 2002). This observation fostered the idea that HA may exert auxin‐like activity, conferring bioactivity that stimulates root expansion, particularly through associations with complex hydrophobic domains (Zandonadi et al. 2007; Canellas et al. 2011). Subsequent evidence supported this view, showing that HA can trigger root induction through auxin‐related mechanisms (Trevisan et al. 2010, 2011), consolidating the concept that these compounds may act as plant growth modulators in a hormone‐like manner.

With the advent of high‐resolution molecular approaches, however, a more complex and multifaceted picture of HS bioactivity has emerged. Transcriptomic analyses in rapeseed ( *Brassica napus* ) using DNA microarrays revealed that HA treatment triggers a broad regulation of metabolic pathways, including stress responses and carbon, nitrogen, and sulfur metabolism (Jannin et al. 2012). More recently, studies in tomato ( *Solanum lycopersicum* L.) demonstrated that vermicompost‐derived HS modulates phenylpropanoid metabolism and defense responses (Scotti et al. 2024). Complementary RNA‐seq studies in maize ( *Zea mays* L.) revealed hormetic responses, in which sub‐stimulatory doses of HA promoted root growth and enhanced antioxidant enzyme activity (Canellas et al. 2020). At the proteome level, HA was found to modulate proteins associated with energy metabolism, nitrogen assimilation, vesicle trafficking, and stress responses, including increased abundance of 2‐cys peroxidase, putative VHS/GAT proteins, and glutathione‐related enzymes (Nunes et al. 2019).

Among the proposed mechanisms, the generation and signaling role of reactive oxygen species (ROS) has emerged as a central element. From the pioneering work of Vaughan and Malcolm (1985), which reported HA‐induced superoxide (O_2_^−^) production and peroxidase regulation, to more recent studies in maize and rice, accumulating evidence indicates that both ROS and nitric oxide (NO) mediate HA‐induced root responses (Zandonadi et al. 2010; Cordeiro et al. 2011, 2017; García et al. 2012, 2014; García, Santos, et al. 2016a). Mechanistically, HA activates NADPH oxidases, enhances ROS production, and stimulates membrane repolarization via H^+^‐ATPase, thereby modulating root growth (Santos et al. 2025). The crosstalk between ROS and hormone signaling was recently confirmed by Souza et al. (2025), who demonstrated, through inhibitor‐based approaches, that HA effects in rice depend on a concerted integration of ROS and hormonal pathways. Additional evidence from Zandonadi et al. (2025) further supports the central role of the RBOH/ROS/auxin/H^+^‐ATPase module in root growth regulation by HA.

Although numerous studies have documented regulatory and physiological effects of HA in plants, a coherent mechanistic framework explaining their mode of action remains missing. A major limitation derives from the intrinsic structural heterogeneity of HAs, which varies substantially according to source material and extraction procedures. This chemical variability has obscured the identification of specific structural features responsible for biological activity and has limited cross‐study reproducibility. While the present study does not attempt to resolve the broader issue of HA heterogeneity, it deliberately employs a chemically characterized HA derived from vermicompost, a source consistently associated with robust biostimulant activity and stress‐protective effects across plant species (Nunes et al. 2019; Silva et al. 2023). By working with defined and structurally well characterized material, we reduce compositional ambiguity and enable more precise mechanistic interpretation.

Beyond compositional variability, a more fundamental limitation in the field concerns the temporal resolution at which HA‐induced responses have been investigated. Most transcriptomic and metabolic studies have examined plants 24 h or several days after treatment, thereby capturing downstream adaptive remodeling rather than the primary molecular events triggered by HA perception. Consequently, current models of HA action are largely inferred from late‐stage correlations, while the early transcriptional hierarchy that initiates redox modulation, hormonal crosstalk, and growth responses remains poorly defined. Without resolving these initial signaling decisions, mechanistic interpretations remain incomplete. Importantly, although the involvement of ROS and hormones such as auxin, ABA, and JA has been previously reported (Nunes et al. 2019; Souza et al. 2022; Santos et al. 2025; Zandonadi et al. 2025; De Hita et al. 2020; Olaetxea et al. 2015; Silva et al. 2023), the sequence, coordination, and early transcriptional integration of these pathways have not been characterized at the onset of HA exposure. Whether HA perception first activates redox signaling modules that subsequently reshape hormonal networks, or whether these pathways are simultaneously reprogrammed, remains unknown.

Here, we address this unresolved question by profiling early transcriptional changes in rice roots following a short‐term (4 h) exposure to a chemically characterized vermicompost‐derived HA. This approach captures the immediate molecular landscape preceding visible morphological or metabolic adjustments, thereby allowing the identification of primary regulatory networks activated upon HA perception. By integrating RNA‐seq analysis with structural characterization of the HAs, this study moves beyond descriptive accounts of late responses and provides a temporally resolved framework for understanding the early molecular mechanisms underlying HA–induced physiological reprogramming in plants.

## 2 Materials and Methods

### 2.1 Extraction and Purification of Humic Acids (HA)

Humic acids (HA) were obtained from a vermicompost produced using bovine manure as the main feedstock. The composting process was carried out at the Integrated Agroecological Production System (SIPA—Fazendinha Agroecológica, km 47), located in Seropédica, Rio de Janeiro, Brazil. After composting, the material was air‐dried at room temperature, sieved for homogenization, and subsequently subjected to humic fraction extraction. Extraction and purification of HA followed the protocol recommended by the International Humic Substances Society (IHSS) and described by Swift (1996). Briefly, humic fractions were extracted by mixing vermicompost with 0.1 mol L^−1^ KOH at a ratio of 10:1 (v/w) under a nitrogen atmosphere, with continuous stirring for 16 h. The supernatant, containing fulvic acids (FA) and HA, was separated from the insoluble residue (humin). HA were precipitated by lowering the pH of the supernatant to 2.0 with gradual addition of 6 mol L^−1^ HCl, followed by 16 h of equilibration and centrifugation at 5000×g for 10 min. The supernatant was collected and stored as the FA fraction. The HA pellet was redissolved in 0.1 mol L^−1^ KOH under an N_2_ atmosphere, treated with 0.3 mol L^−1^ KCl, and centrifuged at 10,000×g for 20 min to remove suspended solids, followed by reprecipitation with 6 mol L^−1^ HCl (pH 1.0, agitation until equilibrium, followed by 16 h resting). The resulting pellet was resuspended in an acid mixture (0.1 mol L^−1^ HCl:HF, 1:1), stirred overnight at room temperature, and the process was repeated until ash content was reduced to < 1%. The purified HA were then transferred to dialysis membranes (SPECTRA/POR7, 1 kDa cutoff) and dialyzed against distilled water until no Cl^−^ ions were detected by an AgNO_3_ test (Swift 1996). Finally, the purified HA were frozen at −80°C and lyophilized for storage.

### 2.2 Structural Characterization of HA by ^13^C NMR Spectroscopy

Solid‐state cross‐polarization magic angle spinning ^13^C nuclear magnetic resonance spectroscopy (^13^C CP/MAS NMR) was performed using a Bruker AVANCE II 400 MHz spectrometer equipped with a 4‐mm narrow MAS probe, operating at a ^13^C resonance frequency of 100.163 MHz. HA samples were packed into zirconia rotors and sealed with Kel‐F caps, and spectra were acquired at a spinning rate of 8 ± 1 kHz. A total of 2048 data points were collected, with the same number of scans, an acquisition time of 34 ms, and a recycling delay of 5 s. The contact time for the ramped cross‐polarization sequence was set to 2 ms. Spectra acquisition and processing were carried out using the Bruker TopSpin version 2.1. Free induction decay (FID) signals were zero‐filled to 4 k points and processed with a line broadening of 70 Hz. Chemical shift regions were defined, integrated, and expressed as the percentage of the total spectral area. The following regions were assigned: alkyl carbon (C_Alkyl_‐H,R): 0–45 ppm; methoxyl and N‐alkyl carbon (C_Alkyl_‐O,N): 45–60 ppm; O‐alkyl carbon (C_Alkyl_‐O): 60–91 ppm; di‐O‐alkyl (anomeric) carbon (C_Alkyl_‐*di*‐O): 91–110 ppm; aromatic carbon (C_Aromatic_‐H,R): 110–142 ppm; O,N‐substituted aromatic carbon (C_Aromatic_‐O,N): 142–156 ppm; carboxyl carbon (C_COO_‐H,R): 156–186 ppm; and carbonyl carbon (C_C=O_): 186–230 ppm. Spectral workup and integration were performed using ACD/Spectrus Processor 2020 1.1 (García, Santos, et al. 2016a; García, Souza, et al. 2016b).

### 2.3 Morphological Characterization of HA by SEM–EDX

The morphological features of HA were analyzed using a high‐resolution scanning electron microscope (SEM) Phenom ProX Desktop (Thermo Fisher) equipped with an energy‐dispersive X‐ray spectroscopy (EDX) detector. Samples were mounted on stubs using carbon tape and cleaned with a nitrogen jet prior to imaging. Micrographs were acquired at 4700× magnification, field width (FW) of 109 μm, operating voltage of 15 kV (spot mode), working distance (WD) of 9.5 mm, and using a BSD Full detector. Elemental composition of the HA was determined by EDX through point analysis, line scans, area scans, and elemental mapping.

### 2.4 Dose–Response Assay of HA in Rice Seedlings

Seeds of *Oryza sativa* L. cv. Nipponbare were surface sterilized in a 2.5% sodium hypochlorite solution with gentle inversion for 15 min, followed by multiple rinses in deionized water. Sterilized seeds were placed on gauze‐based floating supports and germinated in deionized water. Seed germination and seedling growth were carried out in a controlled‐environment chamber under a photosynthetic photon flux density (PPFD) of 318–330 μmol m^−2^ s^−1^, with a 14 h light/10 h dark photoperiod, 70% relative humidity, and day/night temperatures of 28/24°C, at the Department of Soil Science, Federal Rural University of Rio de Janeiro (UFRRJ).

At 3 days after germination (DAG), seedlings were transferred to 1 L plastic pots (four plants per pot) and grown in modified Hoagland and Arnon (1950) nutrient solution at one‐quarter ionic strength (1/4 IS), adjusted to 2 mM total nitrogen (1.5 mM N–NO_3_^−^ and 0.5 mM N–NH_4_^+^), with pH set to 5.8 for better plant adaptation. At 6 DAG, plants were supplied with one‐half ionic strength (1/2 IS) nutrient solution, maintaining the same nitrogen concentration, which was renewed every 3 days. At 12 DAG, to determine the optimal concentration of humic acid (HA), the nutrient solution was replaced with HA‐supplemented solutions at the following concentrations: 0 (control plants), 2, 5, 10, 30, 50, 80, 100, and 150 mg L^−1^ (the concentrations were determined based on a previous experiment conducted by Tavares et al. (2017)). The humic acids were solubilized in a 1 M KOH solution before being dissolved in a nutrient solution. The pH of the final nutrient solution was adjusted to 5.8. At 19 DAG, plants were harvested 3 h after the onset of the light period to minimize diurnal variation. The experiment followed a completely randomized design, with four replicates (each consisting of two pots) per HA concentration (nine doses × four replicates × eight seedlings), totaling 32 seedlings per treatment. Root system architecture was quantified using WinRHIZO software version 2012b (Regent Instruments) at a setting of 600 dpi (dots per inch).

### 2.5 Time–Course Assay of HA‐Induced Transcriptional Regulation in Rice Roots by qRT‐PCR

The time‐course experiment was conducted under the same growth conditions and experimental setup described above. Rice seedlings ( *Oryza sativa* L. cv. Nipponbare) at 19 days after germination (DAG) were transferred to a nutrient solution supplemented with humic acid (80 mg L^−1^), selected based on prior dose–response analyses, while control plants were maintained in a humic acid–free solution. Root tissues were harvested at 4, 8, 24, and 72 h after the onset of treatment. Four biological replicates were used per treatment and time point, each replicate consisting of pooled roots from 16 plants. Samples were immediately frozen in liquid nitrogen and stored at −80°C until further analyses.

Total RNA was extracted as described previously by Gao et al. (2001) using NTES buffer (0.2 M Tris–HCl, pH 8.0; 25 mM EDTA, pH 8.0; 0.3 M NaCl; and 2% SDS). RNA concentration was determined with the Qubit 2.0 fluorometer (Invitrogen) using an RNA‐specific fluorophore. RNA integrity was verified by resolving 3 μL of each sample on 1% agarose gels stained with Gel Red. Subsequently, 0.5 μg of total RNA was treated with DNase I (DNase I Amplification Grade, Invitrogen), and first‐strand cDNA was synthesized using the High‐Capacity RNA‐to‐cDNA Kit (Life Technologies) according to the manufacturer's instructions. The qRT‐PCR was performed on a StepOne Plus Real‐Time PCR System (Applied Biosystems) with Power SYBR Green PCR Master Mix (Applied Biosystems), following the manufacturer's recommendations. To determine the optimal endogenous control, the Normfinder software was used, and the most stable endogenous control was selected. The relative gene expression data were analyzed using the delta–delta comparative Ct method (2^−∆∆*CT*^) using the *OsACT1* (actin1) as endogenous control and transcript levels were normalized to the control (Livak and Schmittgen 2001; Jain et al. 2006). Primers were designed with NCBI Primer‐BLAST (https://www.ncbi.nlm.nih.gov/tools/primer‐blast/) against the RefSeq mRNA database of the *Oryza sativa L. japonica* cultivar group. Transcript levels of 20 genes previously reported to be regulated by humic substances were quantified. Gene identifiers and primer sequences are provided in Table S1.

### 2.6 RNA‐Seq Library Construction

Based on the time‐course analysis, the 4 h exposure was selected as the sampling point for transcriptomic analysis, as it corresponded to the time point showing maximal transcriptional responsiveness in preliminary time‐course analyses, and previously preserved RNA aliquots were used for RNA‐seq library preparation. RNA quantification, quality control, library construction, and sequencing were performed at the Human Genome and Stem Cell Research Center (HUG‐CELL, University of São Paulo, São Paulo, Brazil). RNA concentration was first assessed by spectrophotometry (NanoDrop One, Thermo Fisher Scientific), and purity was verified using absorbance ratios A_260_/A_280_ and A_260_/A_230_. RNA integrity was initially checked by agarose gel electrophoresis and confirmed with the Agilent 4150 TapeStation System. All samples showed RNA Integrity Number (RIN) values between 8 and 10, indicative of high‐quality RNA. The RNA‐Seq libraries were generated from total RNA using poly(A) selection of mRNA (mRNA‐Seq), following the TruSeq RNA Library Prep Kit v2 protocol (Illumina). Sequencing was performed on an Illumina NovaSeq 6000 platform in paired‐end mode (2 × 150 bp), with an average depth of 60 million paired‐end reads per sample.

### 2.7 Bioinformatic Analysis of RNA‐Seq Data

Raw sequencing reads were quality‐checked with fastp (Chen et al. 2018). Reads with Phred quality scores below Q20 and adapter sequences were trimmed, followed by a second round of quality control. Transcript quantification was performed using the software Salmon (Patro et al. 2017), with reads mapped against the *Oryza sativa* ssp. japonica reference transcriptome (IRGSP‐1.0), retrieved from Ensembl Plants release 60, a genome‐centric platform for plant species (Yates et al. 2022). Each sample included two technical replicates, which were merged and treated as a single biological replicate. Gene‐level abundance estimates were obtained with the R package tximport (Soneson et al. 2016), correcting for transcript length bias. To examine global expression patterns, principal component analysis (PCA) was performed on the expression matrix after: (i) filtering out genes with null or extremely low expression (total read counts < 10 across all samples), (ii) normalizing counts by library size, and (iii) applying a variance‐stabilizing transformation to mitigate heteroscedasticity. Differential expression analysis was conducted with the R package DESeq2 (Love et al. 2014), considering genes as differentially expressed when adjusted to *p* < 0.05 (Benjamini–Hochberg correction) and |fold change| > 1.5.

Functional enrichment analysis of differentially expressed genes (DEGs) was performed with g:Profiler (Kolberg et al. 2023) using the Gene Ontology (GO) and Kyoto Encyclopedia of Genes and Genomes (KEGG) databases. Additional enrichment for conserved protein domains (InterPro) and transcription factors (TFs) was performed using annotations from the PLAZA Monocots database (Van Bel et al. 2022) and the R package clusterProfiler (Wu et al. 2021). Overrepresentation was considered significant when adjusted *p* < 0.05 (Benjamini–Hochberg correction), with the entire set of expressed genes (*N* = 30,829) used as the background.

## 3 Results

### 3.1 Structural Characteristics of Humic Acids (HA) Applied to Plants

**FIGURE 1:**
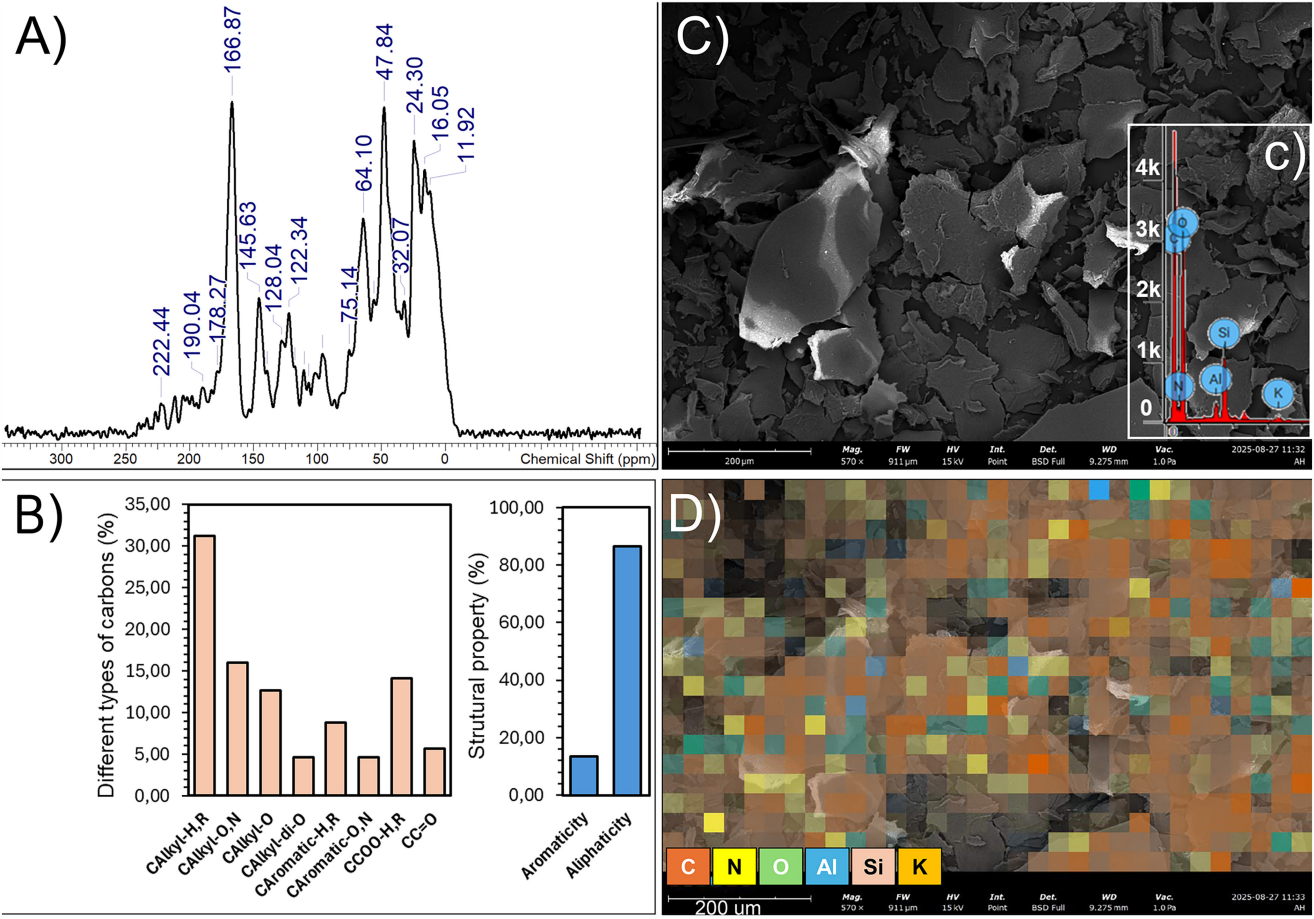
Structural, compositional, and morphological characterization of the humic acids applied to plants. (A) Solid‐state ^13^C NMR CP/MAS spectrum; (B) Relative abundance of carbon types, aliphaticity, and aromaticity of humic acids; (C) SEM micrograph; (c) EDS spectrum; and (D) Surface elemental mapping by EDS.

The solid‐state ^13^C NMR spectrum of the HA used in this study is shown in Figure 1A. The spectrum exhibited well‐defined signals across all regions, confirming the structural heterogeneity of HA isolated from vermicompost. Strong signals between 0 and 45 ppm indicate the presence of low‐functionalized alkyl carbons (C_Alkyl_‐H,R), including methyl and methylene groups (CH_3_‐R, RCH_2_‐R′, and R‐CH‐R′*). These signals mainly correspond to hydrophobic aliphatic fragments such as fatty acid chains, wax residues, cutin, suberin, and microbial lipid remnants. The 45–60 ppm region is attributed to methoxyl carbons (RO‐CH_3_) and α‐carbons of polypeptides (‐CO‐*CHR‐NH), classified as C_Alkyl_‐O,N. These signals may reflect partially preserved lignin fragments (methoxyl groups bound to aromatic rings) as well as nitrogenous residues from microbial proteins or peptides. The 60–90 ppm region contains signals assigned to oxygenated carbons —C—OH, characteristic of cellulose and hemicellulose, as well as lignin‐derived fragments, classified as C_Alkyl_–O. Signals between 90 and 110 ppm are attributed to anomeric carbons of carbohydrates (C_Alkyl_‐*di*‐O), as well as C2 carbons in guaiacyl and syringyl lignin structures. In the 110–160 ppm region, peaks correspond to non‐functionalized aromatic carbons (C_Aromatic_‐H,R), typically associated with C1 atoms of syringyl and guaiacyl lignin units. The 140–160 ppm range includes signals from functionalized aromatic carbons (C_Aromatic_‐O,N). The 160–185 ppm region exhibited intense signals corresponding to carboxylic carbons (—C_COOH_), while the 185–230 ppm range was associated with carbonyl carbons (C_C=O_) of aldehydes and ketones (Keeler et al. 2006; Song et al. 2008; Mao et al. 2011; García, Santos, et al. 2016a; García, Souza, et al. 2016b).

The integration of the ^13^C NMR spectral regions shown in Figure 1A allowed the determination of the relative distribution of carbon functional groups, which is summarized in Figure 1B. Alkyl carbons (C_Alkyl_‐H,R; 0–45 ppm) represented the predominant fraction of total carbon (~30%–32%), indicating a strong contribution of hydrophobic aliphatic domains derived from lipid‐like structures, cuticular materials, and microbial residues. Oxygen‐ and nitrogen‐substituted alkyl carbons (C_Alkyl_‐O,N; 45–60 ppm) accounted for approximately 15%–17%, reflecting the presence of methoxyl groups and peptide‐derived structures. Oxygenated alkyl carbons (C_Alkyl_‐O; 60–90 ppm) contributed around 12%–14%, suggesting the persistence of carbohydrate‐derived moieties such as cellulose and hemicellulose fragments. The di‐O‐alkyl region (90–110 ppm), assigned mainly to anomeric carbons of carbohydrates and specific lignin C2 structures, comprised a smaller fraction (~4%–6%), indicating partial degradation of labile polysaccharides during vermicomposting. Aromatic carbons (110–140 ppm; C_Aromatic_‐H,R) represented approximately 8%–10%, whereas functionalized aromatic carbons (140–160 ppm; C_Aromatic_‐O,N) contributed ~4%–6%, confirming the presence of lignin‐derived aromatic structures, although at lower proportions compared to aliphatic components. Carboxylic carbons (—C_COOH_; 160–185 ppm) accounted for roughly 13%–15% of total carbon, highlighting the oxidized nature of the HA and the abundance of acidic functional groups relevant for cation exchange and metal complexation. Carbonyl carbons (C_C=O_; 185–230 ppm) represented ~4%–6%, indicating minor contributions from ketone and aldehyde structures. When grouped into broader structural domains, aliphatic carbons (0–110 ppm) constituted approximately 80%–88% of total carbon, whereas aromatic carbons (110–160 ppm) represented about 12%–20%. This distribution indicates a predominantly aliphatic character with moderate aromatic condensation, consistent with HA derived from vermicompost and characterized by relatively low humification degree and high biochemical reactivity.

### 3.2 Morphological and Compositional Features of HA Applied to Plants

SEM–EDX analyses (Figure 1C,D) revealed a lamellar morphology with irregular fragments and heterogeneous surfaces, consistent with the structural complexity of humic acids. The EDX spectrum detected C, N, O, Al, Si, and K. Nitrogen was associated with amine/amide groups, oxygen with carbonyl, carboxyl, and hydroxyl groups, Al and Si with incorporated silicate minerals, and K with interactions involving carboxylic and phenolic groups. Elemental mapping confirmed the widespread distribution of C, N, and O, whereas Al and Si were concentrated in specific regions, and K appeared dispersed in isolated spots. These findings highlight the micro‐heterogeneous nature of HA, enriched in oxygenated aromatic groups and with significant potential for ionic complexation.

### 3.3 HA Dose–Response Assay

**FIGURE 2:**
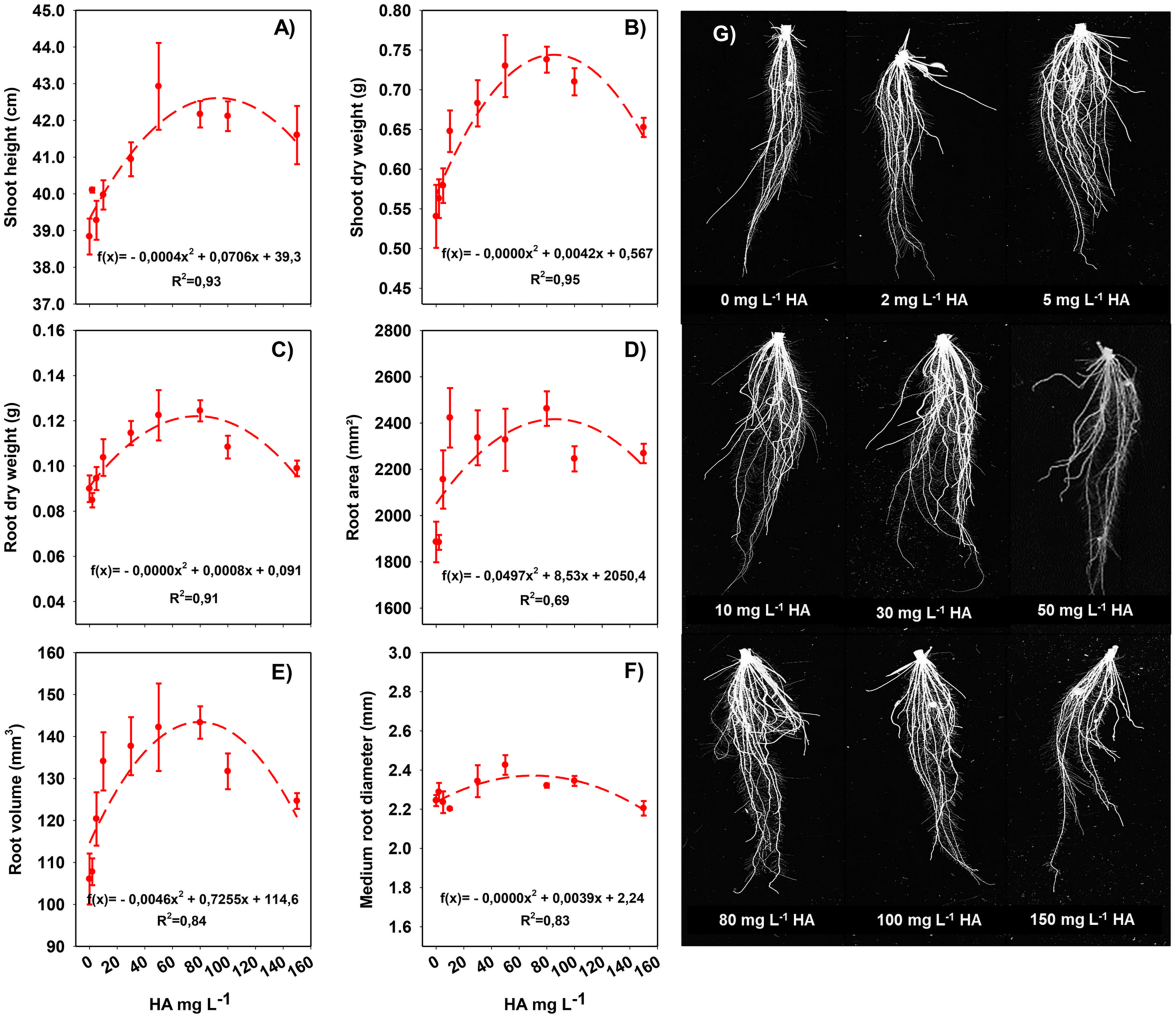
Dose–response assay to determine the humic acid (HA) concentration that maximally promotes rice growth. Tested concentrations were 0, 2, 5, 10, 30, 50, 80, 100, and 150 mg L^−1^ HA. Graphs A–F show shoot height, shoot dry biomass, root dry biomass, root area, root volume, and average root diameter, respectively. Panel G displays roots of representative plants for each treatment. Error bars indicate standard deviation of the mean for 32 plants per dose.

Due to variations in the chemical and physical properties of HA, even when extracted from the same source, a preliminary dose–response assay is essential to identify concentrations most effective in promoting plant development (García, Souza, et al. 2016b). Rice plant morphological parameters exhibited a quadratic response to increasing HA concentrations, characterized by an increase up to an intermediate concentration followed by a decline at higher concentrations (Figure 2A–F). Data on shoot and root biomass (Figure 2B,C), as well as root area and volume (Figure 2D,E), indicate that even low concentrations, such as 5 or 10 mg L^−1^, are sufficient to stimulate growth. This stimulatory effect intensified with higher doses, peaking at approximately 80 mg L^−1^, followed by a decline at the highest concentrations of 100 and 150 mg L^−1^. The image panel (Figure 2G) shows the root systems of representative plants for each treatment, while graphs (Figure 2A–F) depict mean values obtained from the analysis of 32 plants per dose. Based on these results, the 80 mg L^−1^ concentration, which produced the maximal stimulatory effect, was selected for subsequent experiments.

### 3.4 qRT‐PCR Time–Course Assay to Determine the Transcriptional Response of Rice Seedlings to HA

**FIGURE 3:**
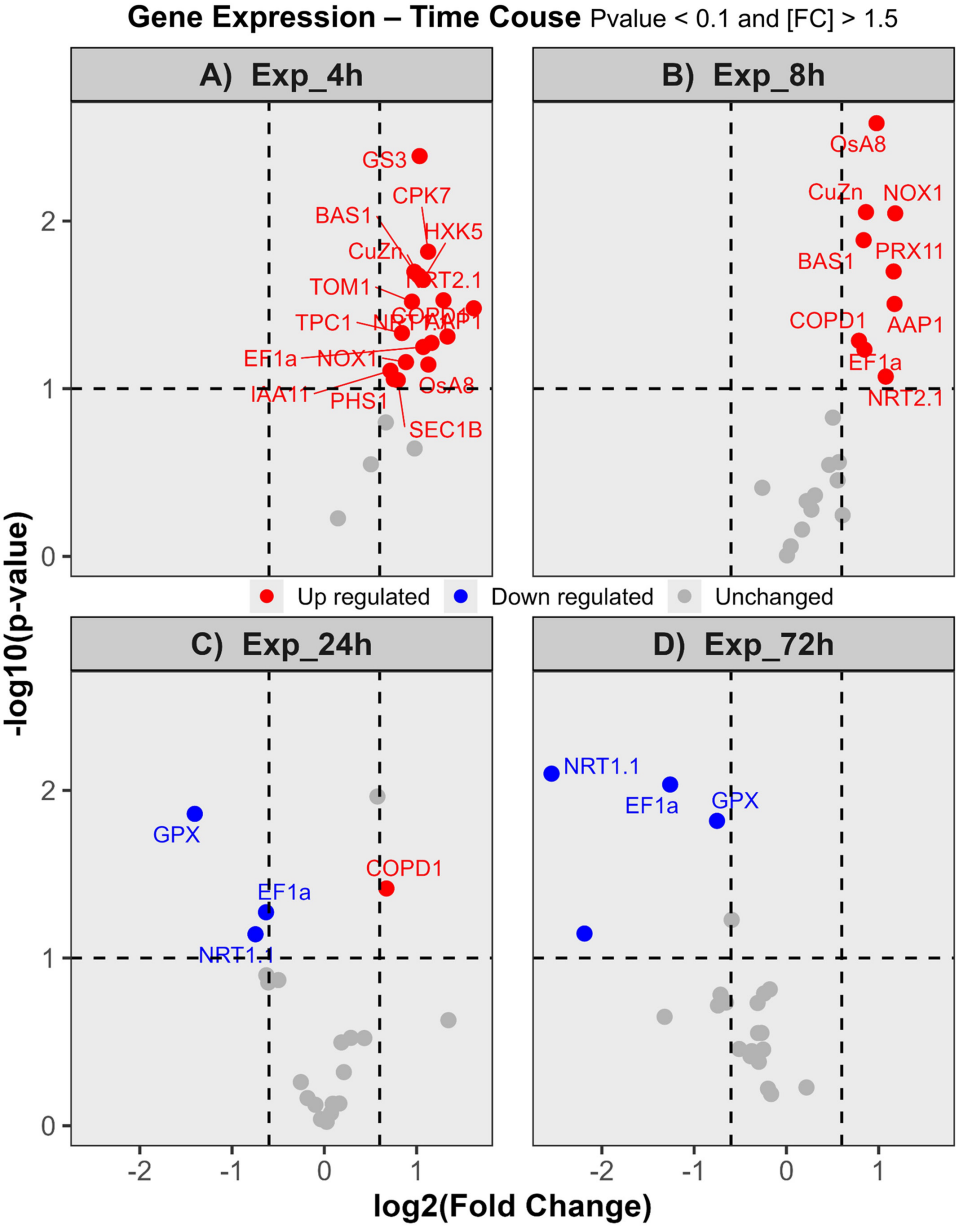
Differential expression of 21 genes (gene descriptions and primer sequences in Table S1) in rice seedlings treated with humic acid (HA) at different time points: 4 h (A), 8 h (B), 24 h (C), and 72 h (D). *OsACT1* (Actin 1) was used as reference genes for normalization. For each collection time, seedlings grown in nutrient solution without HA served as controls.

To evaluate the temporal dynamics of transcriptional regulation in response to HA treatment, a qRT‐PCR assay was performed to quantify the expression of 21 genes previously reported to be HA‐responsive. Descriptions of the genes and primer sequences are provided in Table S1. The selected genes are associated with redox activity, cellular detoxification, stress responses, nutrient transport, and cellular signaling. *OsACT1* (actin 1) was used as a reference gene for normalization. Overall, transcriptional responses to HA were moderate, with induction or repression levels ranging from 1.5‐ to 4‐fold. As shown in Figure 3A, exposure to HA for 4 h resulted in the highest number of regulated genes, with 17 out of 21 analyzed genes being up‐regulated. After 8 h, nine genes remained induced, with no observed repression (Figure 3B). At 24 and 72 h, only four genes were differentially regulated at each time point, predominantly repressed (Figure 3C,D). Based on these results, the 4 h time point was selected for RNA‐Seq analysis, representing the peak of transcriptional activity and likely providing the most informative snapshot of plant perceptions and responses to HA.

### 3.5 Library Quality and RNA‐Seq Analysis

**FIGURE 4:**
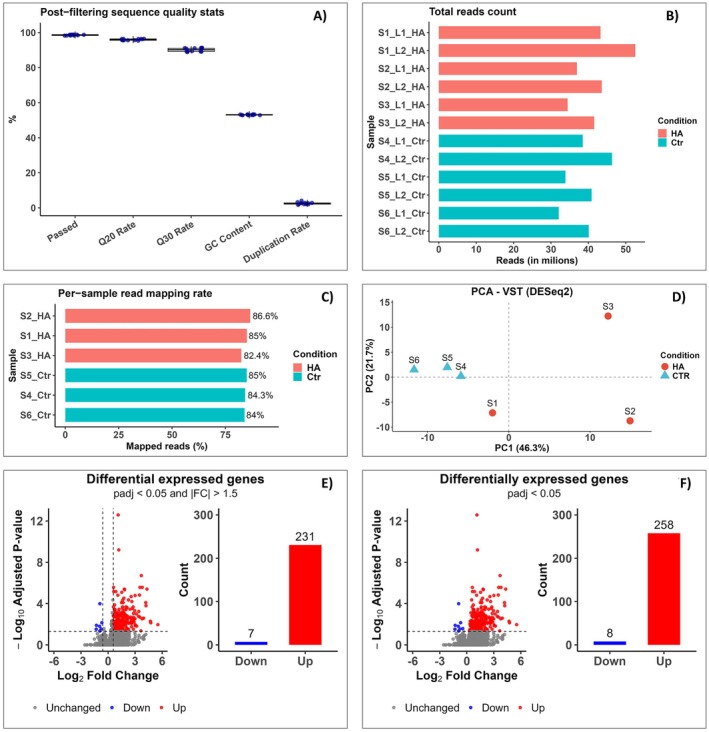
RNA‐Seq statistics of rice seedlings treated with humic acid (HA) for 4 h. (A) Library quality metrics after filtering, expressed as percentages of: (i) reads passing quality filters, (ii) reads with quality ≥ Q20, (iii) reads with quality ≥ Q30, (iv) GC content, and (v) duplication rate; (B) Total reads per biological and technical replicate for each treatment; (C) Mapping rates per biological sample. Reads were mapped to the reference transcriptome using Salmon, with technical replicates considered as a single biological replicate; (D) Dimensionality reduction via principal component analysis (PCA); (E, F) Volcano plots and numbers of up‐ and down‐regulated genes using cutoffs of *P*_adj_ < 0.05 and |FC| > 1.5 (E) or *P*_adj_ < 0.05 only (F).

Aliquots of total RNA preserved from the 4 h time‐point in the previous experiment were used for RNA‐Seq analysis. As shown in Figure 4A, all libraries exhibited high sequencing quality, with nearly 100% of reads passing the minimum quality thresholds in fastp (Chen et al. 2018). Libraries also displayed high proportions of reads with Q20 and Q30 scores, and the GC content showed a narrow unimodal distribution, indicating high library integrity and absence of contamination by exogenous material. Average duplication rates were 2.6%, considered excellent and indicating that the majority of reads were non‐redundant (Figure 4A). The total number of reads was both high and consistent, ranging between 30 and 40 million per analytical replicate, totaling approximately 70 million reads per biological replicate per treatment, underscoring the robustness of the data for transcriptomic analyses (Figure 4B).

Unlike approaches that map reads to a reference genome, we used Salmon (Patro et al. 2017), which maps reads to a reference transcriptome, that is, the complete set of transcripts produced by the genome, including isoforms for each gene, and employs a probabilistic model that allows accurate quantification even for reads mapping to multiple loci. This state‐of‐the‐art approach enables precise transcript quantification, corrects technical biases, and effectively handles paralogous gene families typical of plant transcriptomes. Samples collected 4 h post‐treatment exhibited excellent mapping rates, exceeding 80% (Figure 4C). As expected, principal component analysis (PCA) revealed the formation of two clearly separable groups corresponding to control and HA treatments, despite the presence of one outlier (Figure 4D). Principal component 1 (PC1), accounting for 46.3% of the total variance, clearly discriminated between treatments. At the 4 h time‐point, which likely reflects the moment when plants perceive and respond to HA, the majority of differentially expressed genes were up‐regulated (231), whereas only a few genes were down‐regulated (seven; Figure 4E).

In some cases, many genes are considered differentially expressed when using only the *P*_adj_ < 0.05 filter; however, these genes may not be deemed significant when an effect‐size filter (fold change) is applied. In this experiment, removing the fold change filter would result in very few additional genes being classified as differentially expressed (Figure 4F). For demonstration, we recalculated the number of differentially expressed genes using only *P*_adj_ < 0.05 (Figure 4F). Because the effect is negligible and statistical power is substantially reduced, omitting the fold‐change filter is not recommended. Additionally, HA exerted relatively small effects on the expression of individual genes, with most fold changes below 8 (log2 fold change < 3). Nonetheless, the cumulative effect of these subtle transcriptional changes was sufficient to elicit significant phenotypic responses (Figure 2).

### 3.6 Functional Enrichment Analysis

**FIGURE 5:**
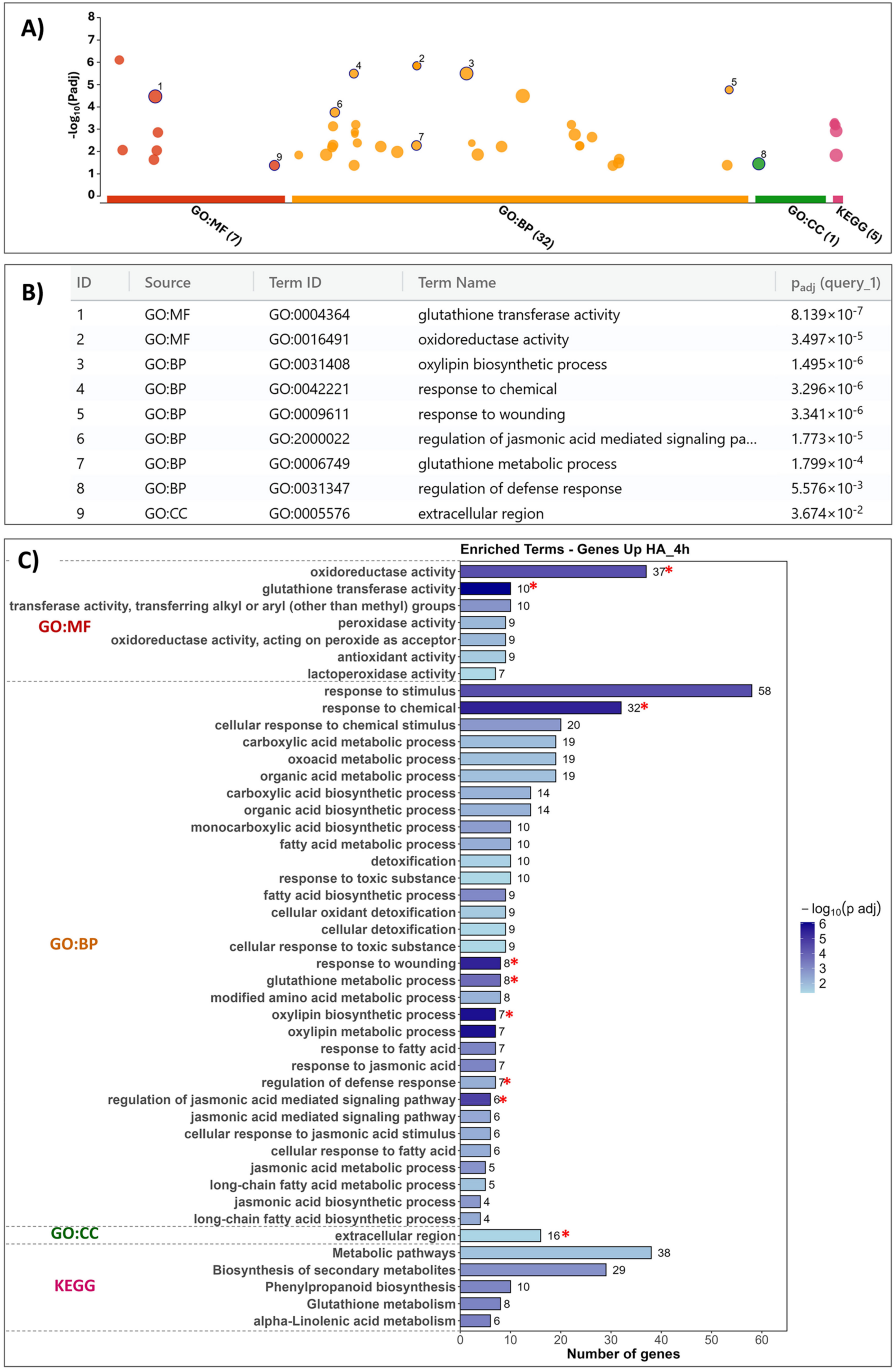
Functional enrichment analysis of up‐regulated genes using the g:Profiler web platform. (A) GOSt plot (Gene Ontology Statistical Test) depicting functionally enriched terms among up‐regulated genes; (B) Highlight of key enriched “driver terms,” representing the most biologically relevant annotations within Gene Ontology; (C) Summary of significantly enriched terms categorized as MF—Molecular Function, BP—Biological Process, CC—Cellular Component, and KEGG—Kyoto Encyclopedia of Genes and Genomes.

The limited number of the seven down‐regulated genes precluded the identification of significantly enriched terms in either GO (Gene Ontology) or KEGG (Kyoto Encyclopedia of Genes and Genomes) categories. In contrast, functional enrichment analysis of the up‐regulated genes (231) revealed numerous significantly enriched terms across GO and KEGG ontologies (Figure 5A–C). Within the GO: Molecular Function (MF) category, the most represented terms included oxidoreductase activity (37 genes), glutathione transferase activity (10 genes), peroxidase activity, antioxidant activity, and lactoperoxidase activity (7–9 genes), reflecting a transcriptional response strongly associated with redox metabolism. A complete list of differentially expressed genes is provided in Table S2.

Among the up‐regulated genes, several were associated with detoxification of reactive oxygen species (ROS) and maintenance of cellular redox homeostasis, indicating a HA‐induced antioxidant response. Notably, genes encoding glutathione S‐transferases (GSTs), including *OsGSTU3/4/5* (Os10g0528400, Os10g0528300, Os09g0367700) and OsGSTF4/5 (Os01g0933900, Os01g0369700), participate in the conjugation of toxic compounds to reduced glutathione (GSH) and the neutralization of lipid peroxides, and are also implicated in defense against oxidative stress or in controlling transient ROS pulses with signaling functions (Moons 2003; Dixon et al. 2010). Within the GSTU subfamily, *OsGSTU3* and *OsGSTU4* are particularly responsive to peroxides, osmotic stress, hypoxia, and heavy metals in rice roots (Moons 2003).

Peroxidase‐encoding genes were also induced, including *OsPRX57* (Os04g0651000), *OsPRX62* (Os04g0688500), and heme peroxidases *OsPRX22*, *OsPRX40*, *OsPRX81*, and *OsPRX83* (Os01g0963000, Os03g0339400, Os06g0522300, Os06g0521500). These enzymes are typically involved in H_2_O_2_ scavenging and cell wall lignification, processes essential for stress defense and adaptive responses. Recent studies have functionally characterized some of these genes: *OsPRX83* is induced by osmotic and oxidative stress, and its overexpression enhances antioxidant responses via peroxidase and APX activity (Bao et al. 2024), while *OsPRX38* mediates increased lignification and reduced arsenic accumulation in transgenic plants (Kidwai et al. 2019). Additionally, *Os1‐CysPrxB* (Os07g0638400), encoding 1‐Cys peroxiredoxin B, an important antioxidant enzyme for peroxide detoxification, was up‐regulated, suggesting a potential role in root system development in rice (Gho et al. 2017).

In the GO: Biological Process (BP) category, enriched terms were predominantly related to responses to stimuli, particularly toxic chemical compounds, with 58 genes annotated under response to stimulus and 32 genes under response to chemical stimulus (Figure 5C). This activation indicates a rapid reprogramming of the plant's adaptive response to humic compounds, consistent with previous reports highlighting the role of HA in activating stress signaling pathways (Castro et al. 2021; Li et al. 2023). Terms associated with glutathione metabolism, such as glutathione metabolic process and cellular oxidant detoxification, were also significantly represented, corroborating activation of the cellular antioxidant system, as noted in the MF category.

Metabolic processes (GO: BP) were enriched for pathways related to carboxylic acid metabolism, phenolic compound biosynthesis, and oxylipin production, including oxylipin biosynthetic process and jasmonic acid biosynthetic/metabolic processes, which are strongly linked to defense signaling and responses to biotic and abiotic stresses (Wasternack and Song 2017). Key genes in the jasmonic acid (JA) biosynthesis pathway were induced, including Allene oxide synthase (*OsAOS2*—Os03g0225900) and 12‐oxophytodienoic acid reductase (*OsOPR1*—Os06g0216300). JA is a central phytohormone mediating biotic and abiotic stress responses, as well as regulating plant growth and development.

Up‐regulation was also observed for *OsMYB21* (Os11g0684000), an R2R3‐MYB transcription factor, previously characterized as *JAmyb*, whose expression is JA‐responsive. Cao et al. (2015) demonstrated that overexpression of *OsJAmyb* in rice enhanced resistance to *Magnaporthe oryzae*, resulting in fewer leaf lesions compared to non‐transgenic controls. Additionally, transcript levels of *OsTIFY11a*, *b*, *c*, and *d* (Os03g0180800, Os03g0181100, Os03g0180900, Os10g0392400) were elevated; these genes belong to the TIFY (JAZ) protein family, which are key regulators of JA signaling. Overexpression of *OsTIFY11b* has been reported to promote plant growth and increase grain weight, likely via JA‐mediated pathways (Hakata et al. 2017).

The C2H2‐type zinc finger transcription factor *OsZFP36* (Os03g0437200) was also induced following humic acid (HA) treatment. This gene is known to be responsive to abscisic acid (ABA) and hydrogen peroxide (H_2_O_2_), playing a key role in antioxidant defense mechanisms. Overexpression of *OsZFP36* in rice has been shown to enhance superoxide dismutase (SOD) and ascorbate peroxidase (APX) activities, contributing to increased tolerance to both water deficit and oxidative stress (Zhang et al. 2014).

Additionally, the expression of *OsCXE3.3* (Os03g0790500) was up‐regulated. This gene is highly responsive to hormonal signals, including ABA and methyl jasmonate (MeJA), as well as major abiotic stresses such as drought, salinity, and cold (Table S2; Zhang et al. 2024). CXE genes, members of the carboxylesterase family, are involved in the modulation of phytohormones such as jasmonic acid (JA) and ABA, regulating their bioactive forms under stress conditions. Therefore, the induction of *OsCXE3.3* suggests its participation in fine‐tuning the active forms of JA and ABA in response to HA stimuli (Zhang et al. 2024).

Ethylene‐responsive genes were also induced, including *OsERF83*, *OsERF91*, and *OsERF124* (Os03g0860100, Os02g0654700, Os12g0168100), which are implicated in both biotic and abiotic stress responses (Table S2). Among these, *OsERF83* is the most extensively characterized, being induced by pathogens such as *Magnaporthe oryzae*, as well as by hormonal treatments with MeJA, ethephon (ethylene), and salicylic acid (SA), indicating its regulation by multiple hormonal pathways during biotic stress (Tezuka et al. 2019). Overexpression of *OsERF83* enhances rice tolerance to blast disease by upregulating a range of pathogenesis‐related (PR) proteins (Tezuka et al. 2019) and has also been reported to confer drought tolerance (Jung et al. 2021).

In the GO: Cellular Component (CC) category, the only significantly enriched term was extracellular region, with 16 annotated genes (Figure 5C). Most of these genes encode peroxidases, some of which overlap with enriched terms in GO: MF. Notably, genes encoding multicopper oxidases, such as *OsLAC1* and *OsLAC10* (Os01g0374600, Os02g0749700), catalyze monolignol oxidation, promoting subsequent polymerization during lignin formation. Their association with the extracellular compartment suggests a role in cell wall–dependent defense responses, particularly via lignin deposition. The plant cell wall represents the first barrier against environmental stress, and reactive oxygen species (ROS) production, often accompanied by increased lignification, is a common response to both biotic and abiotic stress (Liu et al. 2018). Supporting this function, overexpression of *OsLAC10* in *Arabidopsis thaliana* increased root lignification and enhanced tolerance to excess copper (Liu et al. 2017).

KEGG pathway analysis revealed that the regulated genes were primarily associated with metabolic pathways (38 genes), secondary metabolite biosynthesis (29 genes), phenylpropanoid biosynthesis (ten genes), glutathione metabolism (eight genes), and α‐linolenic acid metabolism (six genes). These pathways are crucial for the production of defense‐related compounds, including lignins, flavonoids, and jasmonates, reinforcing the concept that HA acts as a biostimulant, enhancing plant tolerance to biotic and abiotic stresses.

Within the α‐linolenic acid metabolism pathway, induced genes encode key enzymes in the jasmonic acid (JA) biosynthesis pathway, including Allene oxide synthase (*OsAOS2*—Os03g0225900), Oxophytodienoic acid reductases (*OsOPR4*, *OsOPR5*, *OsOPR6*—Os06g0215900, Os06g0215600, Os06g0215500), and 13‐Lipoxygenase (*OsLOX9*). As demonstrated by Wang et al. (2025), *OsLOX9*, a member of the 13‐LOX subfamily, is induced during *M. oryzae* infection, and *lox9* mutants exhibit increased susceptibility, with reduced accumulation of 12‐oxo‐phytodienoic acid, JA, and its bioactive form JA‐Ile; these phenotypes are fully rescued by methyl jasmonate (MeJA) treatment.

The same genes are also represented in the secondary metabolite biosynthesis pathway, which includes additional peroxidases, genes related to fatty acid metabolism, and lignin biosynthesis, such as *OsPAL1* (Os02g0627100), encoding phenylalanine ammonia‐lyase (PAL). PAL catalyzes the conversion of L‐phenylalanine to trans‐cinnamic acid, the first step of the phenylpropanoid pathway leading to lignin, flavonoid, and salicylic acid (SA) synthesis. Overexpression of *OsPAL1* enhances resistance to blast disease, resulting in lesions 3–7 times smaller than in control plants (Zhou et al. 2018).

**FIGURE 6:**
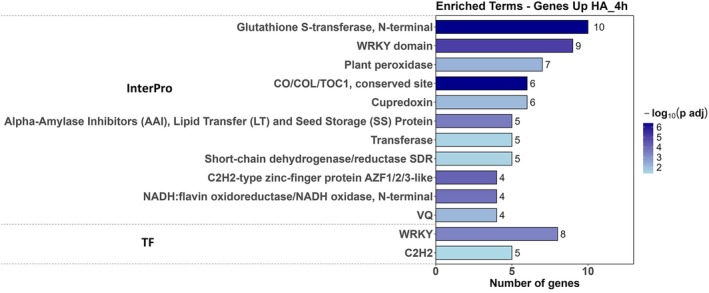
Functional enrichment analysis of up‐regulated genes for InterPro domains and transcription factor (TF) families. Functional annotations were retrieved from the PLAZA Monocots database, and enrichment analysis was performed using the R package clusterProfiler. Terms with fewer than four genes or an intersection size > 60% were removed without affecting the overall interpretation of HA effects. Terms were considered significantly overrepresented at adjusted *p* < 0.05 (Benjamini‐Hochberg correction).

In addition to the enrichments observed in Gene Ontology (GO) terms and KEGG metabolic pathways, analysis of protein domains using the InterPro database and transcription factors (TFs) from the PlantTF Database revealed several significantly overrepresented protein domains and TF families (Figure 6). Within the InterPro category, the most enriched domain was Glutathione S‐transferase, N‐terminal (10 genes), followed by WRKY domain (eight genes) and Plant peroxidase (seven genes). Other relevant domains included Cupredoxin, CO/COL/TOC1 conserved site, and dehydrogenases/reductases, reflecting induction of enzymes involved in antioxidant metabolism and redox stress responses. Regarding transcription factors, enrichment was observed for WRKY TFs (eight genes) and C2H2 zinc‐finger proteins (five genes). The high representation of WRKY TFs suggests modulation of regulatory cascades associated with stress and defense responses, indicating a rapid perception and response of rice seedlings to humic acids (HA), detectable as early as 4 h post‐exposure (Li et al. 2024).

Most WRKY transcription factors identified in this study are linked to the regulation of pathogen defense responses. For instance, *OsWRKY7* (Os05g0537100) was characterized by Zheng et al. (2024), who reported that its expression is induced in rice following inoculation with *Xanthomonas oryzae* pv. oryzae (Xoo). Overexpression of *OsWRKY7* conferred enhanced resistance, whereas knockout plants exhibited increased susceptibility. Similarly, *OsWRKY10* (Os01g0186000) was shown by Wang et al. (2023) to be induced by methyl jasmonate (MeJA) treatment as well as by infection with Xoo and *Magnaporthe oryzae*. *OsWRKY10* functions as a positive regulator of diterpenoid phytoalexin biosynthesis in rice; CRISPR/Cas9 knockout mutants displayed heightened susceptibility to blast, whereas overexpression enhanced disease resistance.

C2H2‐type zinc‐finger transcription factors were also significantly enriched, including *OsZFP15* (Os03g0820400), which is induced by ABA and abiotic stresses. Overexpression of *OsZFP15* increases reactive oxygen species (ROS) production, reducing oxidative stress tolerance but enhancing salt and drought resilience (Wang et al. 2022).

Although not significantly enriched, TFs from the AP2/ERF (APETALA2/Ethylene Responsive Factor) and MYB families were also induced. These TFs are broadly implicated in stress responses, developmental regulation, and hormonal signaling in plants (Xie et al. 2022; Wu et al. 2024; Table S2).

Collectively, the data indicates that HA‐induced redox responses follow a controlled activation of ROS as signaling molecules rather than uncontrolled accumulation typical of deleterious stress. The concomitant induction of multiple genes responsive to both biotic and abiotic stresses, as well as to phytohormones, particularly jasmonic acid (JA) and abscisic acid (ABA), suggests crosstalk between redox and hormonal signaling pathways in the plant response to HA. This pattern resembles a eustress scenario, a moderate form of stress that, unlike excessive stress, can be beneficial by eliciting adaptive responses that enhance plant resilience and stimulate positive physiological processes.

## 4 Discussion

The significant enrichment of genes associated with transferase, oxidoreductase, and antioxidant enzyme activities suggests that HA treatment induces a metabolic reprogramming oriented toward protection against oxidative stress. The induction of genes encoding glutathione S‐transferases and peroxidases, coupled with the enrichment of glutathione metabolism pathways (KEGG), indicates that plants rapidly activate cellular detoxification mechanisms. This observation aligns with previous studies showing that humic substances (HS) can trigger moderate oxidative stress and initiate adaptive responses (Roomi et al. 2018; Souza et al. 2025; Zandonadi et al. 2025). Earlier work has demonstrated that HS, particularly humic acids (HA), modulate the cellular redox state and activate detoxification pathways in plants, as reported by García et al. (2016a) in rice and Nunes et al. (2019) in maize.

The pronounced activation of jasmonic acid (JA)‐related processes, as evidenced by the enrichment of “jasmonic acid biosynthetic process,” “jasmonic acid metabolic process,” and “jasmonic acid‐mediated signaling pathway,” is particularly noteworthy. JA is a key phytohormone orchestrating response to biotic and abiotic stresses, especially wounding, herbivory, and exposure to reactive chemical compounds (Wasternack and Song 2017). The presence of DEGs linked to “response to wounding” (eight genes) and the “oxylipin biosynthetic process” (seven genes), precursors of JA, supports the notion that humic acids act as mild elicitors, activating canonical plant defense pathways.

Another relevant aspect is the induction of fatty acid–related processes, including “fatty acid biosynthetic process,” “monocarboxylic acid biosynthetic process,” and “linolenic acid metabolism” (KEGG). These pathways are critical for the biosynthesis of jasmonates and other volatile signaling compounds (Aslam et al. 2021).

The high number of genes associated with “response to toxic substance,” “detoxification,” “cellular oxidant detoxification,” and “response to oxidative stress” indicates a coordinated mobilization of cellular defense mechanisms. This pattern likely reflects either the reactive nature of humic compounds or the activation of protective pathways even in the absence of actual damage, akin to priming responses (Silva et al. 2023). HA's effect can also be interpreted as a form of eustress (Castro et al. 2021)—a mild stress that stimulates adaptive responses typically associated with biotic or abiotic stress, ultimately enhancing plant performance and resilience.

The enrichment of KEGG metabolic pathways, particularly secondary metabolite biosynthesis such as phenylpropanoids, aligns with the reported induction of phenolic and antioxidant compounds in crops treated with HA, including tomato and wheat (Monda et al. 2021; Lee et al. 2023). These pathways corroborate the present findings, showing that humic substances induce the synthesis of secondary metabolites with defensive and regulatory functions, including flavonoids and lignins, which are derivatives of phenylpropanoid metabolism (Figure 5C). The enrichment of genes associated with the “extracellular region” may relate to modulation of the cell wall and secretion of defense‐ and signaling‐associated proteins.

**FIGURE 7:**
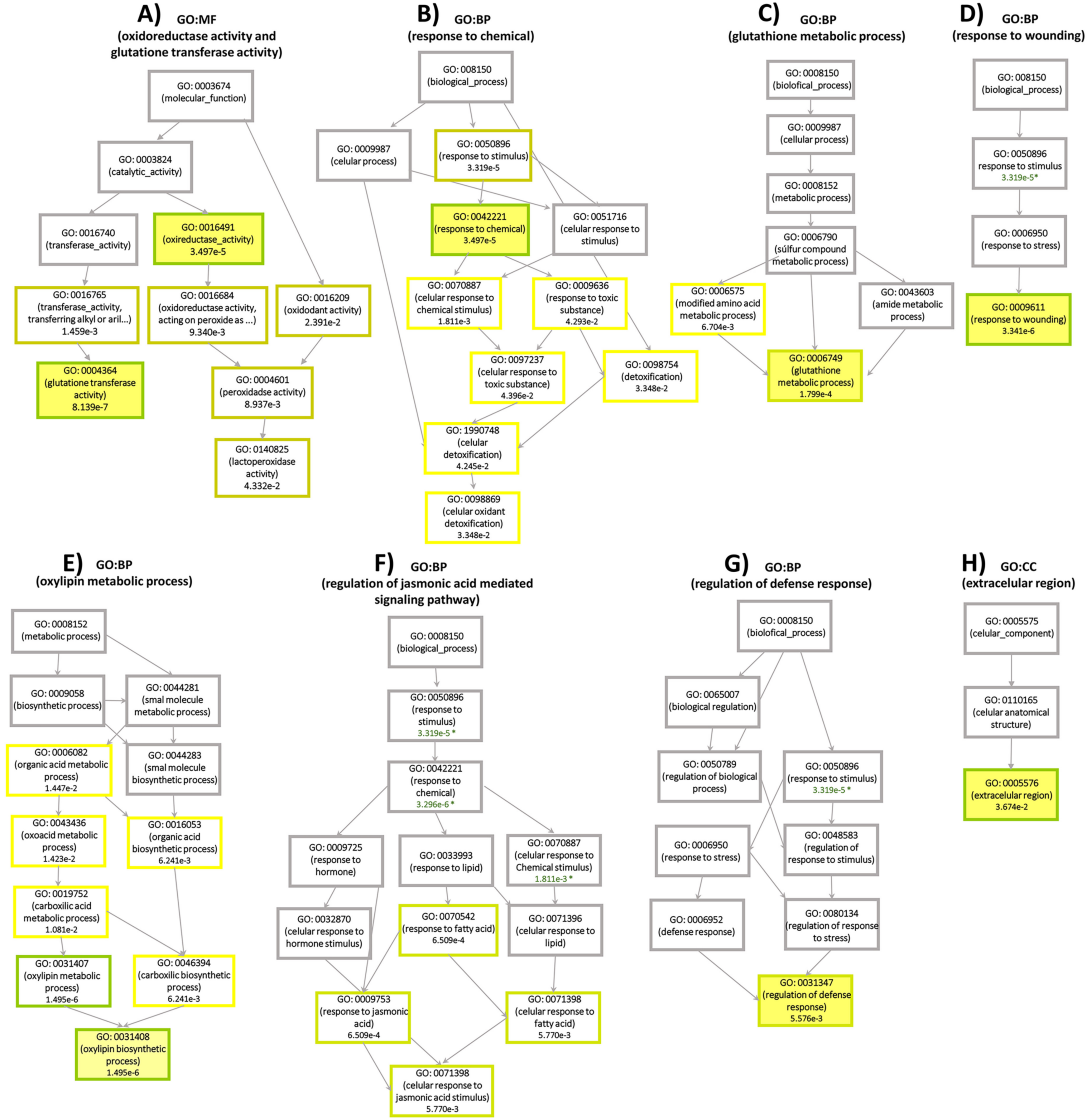
Hierarchical organization of Gene Ontology terms. Molecular Function: (A) oxidoreductase activity and glutathione transferase activity, Biological Process: (B) response to chemical, (C) glutathione metabolic process, (D) response to wounding, (E) oxylipin metabolic process, (F) regulation of jasmonic acid‐mediated signaling pathway, (G) regulation of defense response, and Cellular Component: (H) extracellular region. The significantly enriched and interconnected terms are color‐coded according to their significance.

To better understand the cascade of events underlying plant perception and response to HA, we performed a hierarchical analysis of functionally enriched terms. This approach allows identification of the functional hierarchy from perception to molecular responses triggered by HA treatment (Figure 7). Many significantly enriched terms cluster into interrelated functional branches, highlighting not only the activation of discrete processes but also the coordination of regulatory networks.

In the Molecular Function (MF) domain, the hierarchical organization reveals a progression from “oxidoreductase activity” (GO:0016491) to “peroxidase activity” (GO:0004601) and “glutathione transferase activity” (GO:0004364), indicating activation of pathways for conjugation and neutralization of toxic compounds (Figure 7A). In the Biological Process (BP) domain, a central axis originates from “response to stimulus” (GO:0050896) and branches into “response to chemical” (GO:0042221), culminating in “response to toxic substance” (GO:0009636), reflecting the activation of cellular detoxification mechanisms, as evidenced by the term “cellular oxidant detoxification” (GO:0098869; Figure 7B,C). These functional terms converge on oxidative detoxification processes, consistent with the observed transcriptional pattern.

Within GO:BP, a branch extends through “response to wounding” (GO:0009611), “response to jasmonic acid” (GO:0009753), “response to fatty acid” (GO:0070542), and “cellular response to jasmonic acid stimulus” (GO:0071396), representing a coordinated response via hormonal signaling and oxylipin metabolism, precursors of jasmonic acid (Figure 7D–G). The hierarchical convergence of stimulus response, hormonal signaling, and detoxification enzyme activities underscores HA's systemic action, encompassing signal perception, transcriptional reprogramming, and cellular adaptation to chemical, predominantly oxidative, stress.

Beyond the enrichments observed in Gene Ontology (GO) terms and KEGG metabolic pathways, protein domain analysis using the InterPro database further highlighted the significant representation of protein families involved in oxidative stress responses, plant defense, and transcriptional regulation. The activation of genes harboring glutathione S‐transferase (GST) domains reinforces the hypothesis that humic acids elicit a metabolic “alert” state, promoting the activation of cellular detoxification mechanisms based on glutathione conjugation (Dixon et al. 2010).

The enrichment of transcription factors (TFs) from the WRKY family suggests that HA triggers transcriptional reprogramming mediated by these key regulators, which recognize W‐box promoter elements and primarily control defense‐related genes (Rushton et al. 2010; Zhang et al. 2023). WRKY TFs are also well known to participate in responses to abiotic stresses, including salinity, temperature fluctuations, and nutrient deficits (Li et al. 2024; Ma and Hu 2024), while interacting with hormonal signaling pathways such as ABA and jasmonate (Wasternack and Song 2017). The recurrent identification of the WRKY domain, both in InterPro annotations and among transcription factors, underscores the central role of this family in orchestrating molecular responses to HA. This functional convergence indicates not only the structural presence of the WRKY domain but also the induction of its active forms, which likely act directly in the transcriptional regulation of HA‐responsive genes.

Additionally, the activation of C2H2‐type zinc‐finger transcription factors complements this regulatory network, suggesting the involvement of multiple gene control pathways. These TFs are broadly associated with developmental processes, responses to environmental stimuli, and adaptation to abiotic stresses, as previously described (Jiang et al. 2014). Collectively, these findings indicate that HA treatment initiates a transcriptional response within functional domains associated with detoxification and transcriptional regulation, predominantly via WRKY TFs, potentially enhancing the adaptive capacity of rice under environmental stress conditions.

**FIGURE 8:**
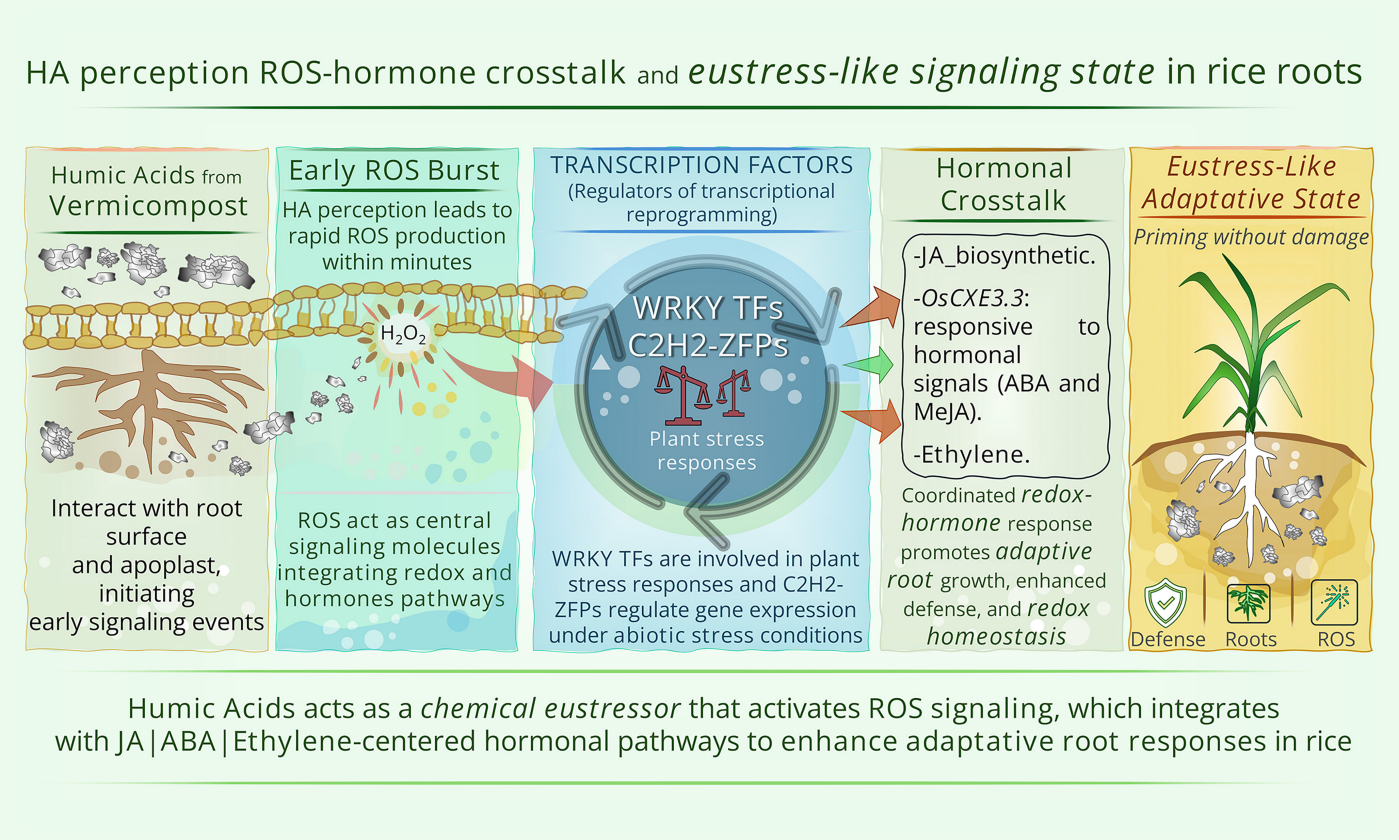
Conceptual model of early perception and signaling triggered by vermicompost‐derived HA in rice plants. The model illustrates the proposed early events underlying rice root responses to vermicompost‐derived humic acids (HA). Upon contact with the root surface, HA is perceived at the apoplast–plasma membrane interface, triggering rapid activation of membrane‐associated processes, including the stimulation of reactive oxygen species (ROS) production. This transient ROS burst acts as a primary signaling component, functioning as a eustress‐like signal rather than a damaging oxidative event. ROS signaling integrates with hormonal pathways, particularly jasmonic acid, as well as abscisic acid and ethylene, promoting hormone crosstalk and the activation of transcription factor–centered regulatory networks. These transcription factors act as key molecular hubs, translating early ROS–hormone signals into coordinated transcriptional reprogramming and downstream metabolic adjustments that support root growth, developmental plasticity, and enhanced stress preparedness.

The results obtained here fulfill the primary objective of this study. We demonstrate that HA applied to rice plants initiates their biostimulant effects within the first 4 h of treatment. Our findings reveal that HA triggers a highly early functional reprogramming, characterized by the coordinated enrichment of Gene Ontology terms associated with responses to chemical stimuli, redox‐related processes, and defense pathways as early as 4 h after treatment. Notably, there is a significant activation of molecular functions related to oxidoreductase and glutathione transferase activities, together with biological processes linked to glutathione metabolism and cellular detoxification, indicating an immediate adjustment of cellular redox status and antioxidant capacity. In parallel, enrichment is observed for pathways associated with oxylipin metabolism and the regulation of JA–mediated signaling, as well as terms related to wound response and regulation of defense responses, suggesting that HA is rapidly perceived as bioactive signals capable of modulating networks typically involved in adaptive and eustress responses. The hierarchical organization of these terms, including extracellular components, supports the hypothesis that HA acts early at the perception–signaling interface, promoting the integrated activation of defense, detoxification, and lipid signaling mechanisms well before the time frames traditionally reported in the literature for HS–induced responses (Figure 8).

The results obtained here advance the establishment of a mechanistic framework for the mode of action of humic substances (HS) in plants in two fundamental directions. The early regulation of 231 induced genes associated with multiple signaling pathways integrated within a ROS–hormone crosstalk network demonstrates that vermicompost‐derived humic acids (HA) possess structural features capable of biostimulating plant metabolism in a manner consistent with HA from distinct sources. This is particularly significant because comparable effects have been consistently reported across different origins and plant species. In maize, HA extracted from vermicompost regulate both hormonal and redox signaling pathways (Nunes et al. 2019; Souza et al. 2022), while HA obtained from oxidized sub‐bituminous coal stimulate root growth in Arabidopsis and maize ( *Zea mays* ) through modulation of RBOH‐dependent ROS signaling (Santos et al. 2025; Zandonadi et al. 2025). Similarly, leonardite‐derived HA were shown to involve ABA as a mediator of physiological responses in cucumber ( *Cucumis sativus* L.) (Olaetxea et al. 2019), and HA and fulvic acids (FA) derived from coal and peat exert protective effects in wheat ( *Triticum aestivum* L.) under water deficit (Kulikova et al. 2018). Collectively, and in agreement with Nardi et al. (2021) and García et al. (2016b), these findings support the inference that HA share a conserved structural identity independent of their source, conferring the chemical properties required to define their function as plant biostimulants.

Conversely, substantial evidence supports the involvement of a robust hormonal regulatory network underlying the physiological effects of HA in plants. De Hita et al. (2020) demonstrated that HS promote beneficial responses in cucumber ( *Cucumis sativus* L.) through a transient mild‐stress mechanism mediated by JA and its bioactive conjugate JA‐Ile. Similarly, Silva et al. (2023), using peat‐ and vermicompost‐derived HA, reported JA‐dependent regulation associated with enhanced protective responses in tomato ( *Solanum lycopersicum* L.). In addition, Olaetxea et al. (2015), employing the same HA–plant experimental framework, identified the involvement of abscisic acid (ABA)‐mediated pathways in the promotion of root growth. Earlier, Maffia et al. (2025) demonstrated that auxin signaling pathways play a central role in mediating HS‐induced physiological responses across different plant species.

Although these studies were reported independently and focused on distinct hormonal branches, our results provide evidence for a functional convergence among these pathways. We show that the effects of HS in plants are mechanistically connected through an early redox‐regulatory layer involving ROS signaling. In this framework, HA‐induced modulation of cellular redox status appears to generate a transient ROS signal that acts as a messenger, integrating and fine‐tuning downstream hormonal pathways, including JA‐, ABA‐, and ethylene‐dependent signaling. Thus, redox regulation and ROS‐dependent crosstalk with hormone signaling constitute an initial signaling hub from which the previously described physiological outcomes, such as enhanced growth, improved stress tolerance, and metabolic reprogramming, are orchestrated.

## Author Contributions

This study is a collaborative effort of all authors. L.A.S, J.N.O.S., A.C.G., and A.F.F.S. conceptualized and designed the experiments. J.N.O.S., R.V.S., I.A.P.C., and O.C.H.T. performed the experiments and conducted data analysis. J.N.O.S., A.C.G., A.F.F.S., and L.A.S. wrote the original draft and finalized the manuscript. C.C.L.O., A.L.S.P.N., and R.V.S. contributed to specific experiments and measurements. L.A.S., R.L.L.B., and M.S.F. provided resources and project support. L.A.S. and A.C.G. supervised the research and performed critical review and editing of the manuscript. All authors read, contributed to, and approved the final manuscript.

## Funding

This work was supported by the Rio de Janeiro Research Foundation (FAPERJ) and the National Council for Scientific and Technological Development (CNPq). The article processing charge (APC) was funded by the Coordination for the Improvement of Higher Education Personnel (CAPES).

## Supporting information


**Table S1:** List of genes analyzed in the time‐course response assay to humic acid (HA).
**Table S2:** Differentially expressed genes from the RNA‐Seq experiment.

## Data Availability

All data supporting the findings of this study are available within the manuscript and its Supporting Information files.
