# Supplementary material for: Humic Acid Induces an Adaptive Stress Response During Early Signaling in Rice
Source: Physiol Plant. 2026 Apr 19;178(2):e70877. doi: 10.1111/ppl.70877 (PMC13092416; doi:10.1111/ppl.70877)
Supplement: Supplementary file 1 — Table S1: List of genes analyzed in the time‐course response assay to humic acid (HA). Table S2: Differentially expressed genes from the RNA‐Seq experiment. [file PPL-178-e70877-s001.docx]

**SUPPORTING INFORMATION**

**Humic Acid Triggers a Eustress-Like State during Early Signaling in Rice**

José Nivaldo de Oliveira Sátiro^1^; Andrés Calderín García^2,3^; Andressa Fabiane Faria de Souza^1^; Clenya Carla Leandro de Oliveira^1^; André Luís da Silva Parente Nogueira^1^; Rômulo Vallim dos Santos^1^, Inês Ariane de Paiva Câncio^1^; Orlando Carlos Huertas Tavares^2^; Ricardo Luiz Louro Berbara^2,3^; Manlio Silvestre Fernandes^1^; Leandro Azevedo Santos^1,3*^

**Affiliations**

^1^ Plant Nutrition Laboratory, Department of Soils, Federal Rural University of Rio de Janeiro (UFRRJ), Seropédica 23890-000, RJ, Brazil

^2^ Soil Biological Chemistry Laboratory, Department of Soils, Federal Rural University of Rio de Janeiro (UFRRJ), Seropédica 23890-000, RJ, Brazil

^3^ NIBBA – Biotechnology Innovation Hub for Agricultural Bioinputs, Federal Rural University of Rio de Janeiro, Brazil.

***Corresponding author**

Correspondence to Leandro Azevedo Santos: azevedo.ufrrj@gmail.com

Table S1. List of genes analyzed in the time-course response assay to humic acid (HA).

| **Genes** | **Locus RAP*** | **Gene symbol** |  |
| --- | --- | --- | --- |
| chloroplast 2-Cys peroxiredoxin BAS1 | Os02g0537700 | BAS1 | F: GTCTGCCCGACCGAGATTAC  R: CCAAGCCCACCAGATTTCCT |
| glutathione peroxidase 3 | Os02g0664000 | GPX | F: CTGCGTTGCATTGAGCACTT  R: GGGGCAAAGTGATGCAGTAAG |
| cytosolic glutamine synthethase 1;2 | Os03g0223400 | GS3 | F: TACGCCGGGATCAACATCAG  AACCCACACTTGGTCAGCAG |
| class III peroxidase 112 | Os07g0677300 | PRX112 | F: ACATCTCGCCCTTGACTGGA  R: CAATGTCGGGGCCTAGCTTT |
| Cu/Zn-Superoxidase Dismutase1 | Os03g0351500 | CuZnSOD1 | F: AGATTCCAAACCAGCAGGAGT  R: AAGCACAACAACAGCCTTCAC |
| Target of rapamycin - Serine/threonine (Ser/Thr) protein kinase | Os05g0235300 | TOR | F: GGCTGAATGGATGAGGCACT  R: CTCACGACCAACAGAAGGCT |
| Similar to Sec1p-like protein 2 | Os06g0135900 | SEC1B | F: CGAAGCTGAAGATGCTAACTGC  R: CGTAGCAATGGGTTTCTTACCAA |
| Hexokinase 5 | Os05g0522500 | HXK5 | F: CAAGATGCCAAAAGACGGCA  R: CCACCGAAGACGAGACATCA |
| TOM1-like protein 9 | Os01g0229200 | TOM1 | F: TACAGCAACAGCCAAGGGAG  R: TGTAGAGTAGGGCGAGCTGT |
| rice elongation factor 1A (eEFIA)-3 | Os03g0177900 | EF1a | F: ACTGATGCTCTGAGTCCGAT  R: AGCAGGGAATAGCACAAACGA |
| coatomer subunit delta protein 1 | Os05g0310800 | DELTA-COP1 | F: CTGGAATCCGTCCTCTGAAGG  R: GTGAGGCAACAAACATCCCT |
| plasma membrane H^+^-ATPase 7 | Os04g0656100 | OsA7 | F: TCCAACACGCTCTTCAACGA  R: CCTTCAGCTTCACCACCGAT |
| indole-3-acetic acid inducible11 | Os03g0633500 | AIA11 | F: AGCTGAGAGATGACCTGGAGT  R: AAGGCCAATGGCTTCAGACC |
| nitrate transporter 1.1B | Os10g0554200 | OsNRT1.1 | F: AGGGGGTACGTCTACAAGGA  R: AGCTTGCGTATGTCATGCTG |
| high-affinity nitrate transporter 2.1 | Os02g0112100 | OsNRT2.1 | F: CCACGGTGCAAGTCTCAAGT  R: GTCGCAGAATTGTTTACGCCT |
| calcium-dependent protein kinase 7 | Os03g0128700 | OsCPK7 | F: TAGCCCTCGAAGAAGCAAGG  R: TCCCCAACCTTACGAGACCT |
| Two pore calcium channel protein 1 | Os01g0678500 | OsTPC1 | F: CGGAGCCCACTGTTTGAGTA  R: TCTTGCCACACTTTCTGCGA |
| Protein phosphatase 3 | Os01g0311500 | OsPHS1a/OsPP3 | F: TTACCAAGGCGTTCGCTGAT  R: ACATCACCACACTGCCACAA |
| respiratory burst oxidase homolog B | Os01g0360200 | OsNox1/OsRbohB | F: CGATGCTCGTTCTGCTCTCA  R: TAGGTCGTGCGAAATGGGTC |
| Respiratory Burst Oxidase Homolog H | Os12g0541300 | OsNox9/RbohH | F: TACTTCGGGCAGACACGGAT  R: GCGGGTTGCTGTCACTAAG |
| plasma membrane H^+^-ATPase 8 | Os03g0100800 | OsA8 | F: TGTTTAACCTACAACACGACAATGC  R: AATGGGATGGGAAAGGAAAATAC |
| Ubiquitin 5 | Os01g0328400 | OsUBQ5 | F:ACCACTTCGACCGCCACTACT  R: ACGCCTAAGCCTGCTGGTT |
| Actin 1 | Os03g0718100 | OsACT1 | F:CTTCATAGGAATGGAAGCTGCGGG  R: CGACCACCTTGATCTTCATGCTGC |

*The Rice Annotaion Project Database (RAP-DB). https://rapdb.dna.affrc.go.jp

**Table S2**. Differentially expressed genes from the RNA-Seq experiment.

| **gene** | **description** | **Oryzabase Gene Symbol Synonym(s)** | **log2FoldChange** | **FoldChange** | **pvalue** | **padj** | **contrast** | **regulation** |
| --- | --- | --- | --- | --- | --- | --- | --- | --- |
| Os08g0374701 | Similar to Protein kinase domain containing protein, expressed. (Os08t0374701-01) | OsRLCK253 | 5.529292403 | 46.18307734 | 7.37E-05 | 0.010967 | 4h | Up |
| Os07g0526600 | Alpha/beta hydrolase fold-3 domain containing protein. (Os07t0526600-01) | OsCXE7.7 | 4.738232597 | 26.69009615 | 4.00E-05 | 0.007285 | 4h | Up |
| Os07g0590100 | Zinc finger, C2H2 domain containing protein. (Os07t0590100-01) | OsDLN193, OsZOS7-09 | 4.691770763 | 25.84423803 | 2.38E-05 | 0.005113 | 4h | Up |
| Os01g0714800 | WRKY transcription factor 26, Response to M. oryzae infection (Os01t0714800-01) | OsWRKY26, OsWRKY59, DLN19, OsDLN19 | 4.316641731 | 19.92684954 | 1.36E-09 | 4.03E-06 | 4h | Up |
| Os02g0749700 | Laccase, Lignin biosynthesis, Response to Cu stress of rice roots (Os02t0749700-01) | OsLAC10 | 4.206637355 | 18.46392489 | 1.86E-07 | 1.63E-04 | 4h | Up |
| Os11g0643100 | Tryptamine benzoyl transferase 2 (Os11t0643100-01) | Os-TBT2, OsTBT2 | 4.182204731 | 18.15386377 | 8.24E-08 | 8.12E-05 | 4h | Up |
| Os08g0374600 | Similar to Receptor kinase-like protein. (Os08t0374600-00) | OsRLCK253 | 3.93978656 | 15.34595538 | 9.23E-06 | 0.0026 | 4h | Up |
| Os10g0380800 | Similar to Tryptophan decarboxylase. (Os10t0380800-00) | OsTDC6 | 3.795086972 | 13.8814558 | 6.97E-10 | 2.75E-06 | 4h | Up |
| Os07g0526400 | Polyketide synthase, type III domain containing protein. (Os07t0526400-01) | OsPKS15, OsCHS15, CHS15 | 3.773415156 | 13.67449031 | 1.11E-05 | 0.003012 | 4h | Up |
| Os11g0340477 | Zinc finger, C2H2-type domain containing protein. (Os11t0340477-01) |  | 3.691371838 | 12.91854637 | 2.52E-11 | 1.98E-07 | 4h | Up |
| Os01g0374600 | Similar to Laccase-21. (Os01t0374600-00) | OsLAC1 | 3.647181442 | 12.52884434 | 4.50E-04 | 0.043812 | 4h | Up |
| Os03g0437200 | C2H2-type zinc finger protein, Abscisic acid-induced antioxidant defence, Water stress and oxidative stress tolerance (Os03t0437200-01) | ZFP36, OsZFP36, OsBSRD1, Bsr-d1, BSR-D1, DLN91, OsDLN91, OsZOS3-12 | 3.539198362 | 11.6253187 | 6.34E-10 | 2.75E-06 | 4h | Up |
| Os12g0168100 | Similar to AP2 domain containing protein RAP2.6 (Fragment). (Os12t0168100-01);Drought-responsive ethylene response factor 8, ERF family protein (Os12t0168100-02) | DERF8, OsDERF8, OsERF#124, OsERF124, AP2/EREBP#158, AP2/EREBP158 | 3.52698621 | 11.5273278 | 6.75E-06 | 0.002074 | 4h | Up |
| Os01g0389700 | Protein of unknown function DUF679 family protein. (Os01t0389700-01) |  | 3.491444663 | 11.24681553 | 8.32E-09 | 1.51E-05 | 4h | Up |
| Os03g0860100 | Ethylene response factor, Transcriptional activator, Positive regulation of disease resistance (Os03t0860100-01) | OsERF#083, OsERF083, OsERF83, AP2/EREBP#163, AP2/EREBP163 | 3.409351022 | 10.62470605 | 1.10E-06 | 5.92E-04 | 4h | Up |
| Os09g0368200 | Polyamine oxidase 6, Activation of herbivore-induced polyamine catabolism, Positive regulation of rice defense against BPH (Os09t0368200-01) | PAOb, OsPAOb, OsPAO6, OsPAO2, PAO6, PAO2 | 3.386854223 | 10.46031377 | 5.13E-06 | 0.001733 | 4h | Up |
| Os10g0515900 | Cytochrome P450 family protein. (Os10t0515900-01) | OsCYP89B1 | 3.176475401 | 9.040956424 | 1.16E-04 | 0.015226 | 4h | Up |
| Os03g0674900 | Regulation of resistance to bacterial leaf streak (Os03t0674900-01) | OsRBLS3 | 3.137012466 | 8.797005208 | 2.42E-06 | 0.001022 | 4h | Up |
| Os07g0564500 | Pyridine nucleotide-disulphide oxidoreductase, NAD-binding region domain containing protein. (Os07t0564500-01) | OsNDA2 | 3.028521078 | 8.159728093 | 7.94E-05 | 0.011661 | 4h | Up |
| Os11g0582000 | Hypothetical conserved gene. (Os11t0582000-00) |  | 3.022236042 | 8.124257923 | 1.03E-04 | 0.014113 | 4h | Up |
| Os10g0469600 | LRR-receptor-like protein (LRR-RLP) family protein (Os10t0469600-01) |  | 3.004367638 | 8.024256028 | 1.05E-08 | 1.66E-05 | 4h | Up |
| Os04g0179700 | 9-beta-pimara-7,15-diene synthase, Momilactone phytoalexins biosynthesis, Defense response (Os04t0179700-01) | OsKS4, KS4, KSL4, OsKSL4, DTS2, OsDTS2, OsTPS16, TPS16, TPS8, OsTPS8 | 2.984898082 | 7.916693961 | 1.09E-05 | 0.003012 | 4h | Up |
| Os10g0393800 | Lipase, GDSL domain containing protein. (Os10t0393800-01) | OsGELP105 | 2.961138027 | 7.787379999 | 8.52E-07 | 4.91E-04 | 4h | Up |
| Os05g0104700 | Leucine-rich repeat (LRR) protein, Inhibitor of fungal polygalacturonase, Defence response (Os05t0104700-01) | Ospgip4, pgip4, OsPGIP4 | 2.885899256 | 7.3916644 | 3.21E-05 | 0.006219 | 4h | Up |
| Os07g0587300 | Hypothetical conserved gene. (Os07t0587300-01) |  | 2.837857945 | 7.149577282 | 1.15E-05 | 0.00305 | 4h | Up |
| Os11g0244100 | Transferase family protein. (Os11t0244100-00) |  | 2.821439187 | 7.06867194 | 1.95E-06 | 9.03E-04 | 4h | Up |
| Os10g0391400 | Jasmonate ZIM-domain (JAZ) protein, TIFY family protein, Negative regulation of JA signal transduction pathway, Activation of hypersensitive cell death (Os10t0391400-01) | OsJAZ 13, OsJAZ13, JAZ13, OsTIFY11e, OsJAZ13a, OsJAZ13b, OsJAZ13c, OsJAZ13d | 2.815211774 | 7.038225676 | 6.98E-06 | 0.002115 | 4h | Up |
| Os09g0367700 | Tau class glutathione S-transferase, Tolerance against sheath blight disease (Os09t0367700-01) | OsGSTU5 | 2.80107089 | 6.969575995 | 2.71E-04 | 0.030109 | 4h | Up |
| Os01g0668901 | S-Domain receptor like kinase-2, Response to submergence, Response to bacterial and fungal infection (Os01t0668901-00) | SDRLK-2, OsSDRLK-2, OsSDRLK2 | 2.774206471 | 6.840996403 | 2.81E-07 | 2.14E-04 | 4h | Up |
| Os04g0180400 | Similar to Cytochrome P450 99A2. (Os04t0180400-01) | OsCYP99A2 | 2.767197782 | 6.807843074 | 2.69E-05 | 0.005536 | 4h | Up |
| Os01g0963000 | Similar to Peroxidase BP 1 precursor. (Os01t0963000-01);Similar to Peroxidase BP 1 precursor. (Os01t0963000-04) | prx22, OsPRX22, OsPOD | 2.708522194 | 6.53651744 | 4.41E-07 | 3.07E-04 | 4h | Up |
| Os03g0676400 | VQ domain containing protein. (Os03t0676400-01) | OsVQ13 | 2.64147829 | 6.239707021 | 5.66E-08 | 6.38E-05 | 4h | Up |
| Os12g0635400 | VQ domain containing protein. (Os12t0635400-01) |  | 2.627592837 | 6.179940025 | 1.21E-05 | 0.00314 | 4h | Up |
| Os11g0642400 | Tryptamine benzoyl transferase 1 (Os11t0642400-01) | Os-TBT1, OsTBT1 | 2.621215883 | 6.152683936 | 4.95E-05 | 0.00831 | 4h | Up |
| Os08g0140300 | Aromatic L-amino acid decarboxylase (AADC), Senescence-induced serotonin biosynthesis (Os08t0140300-01) | OsTDC, TDC, TDC2, OsTDC1, OsTDC4 | 2.616039102 | 6.130645999 | 4.54E-05 | 0.007834 | 4h | Up |
| Os08g0508800 | Type 2 13-lipoxygenase, Herbivore-induced defense response (Os08t0508800-01) | Lox2:Os:1, OsLOX9, LOX9, OsLOX8, LOX8, OsLOX2, LOX2, OsRLL, OsHI-LOX, HI-LOX, RLL2 LOX, RLL2, Lox2osPil, OsLOX2;1, LOX2;1, OsLOX1, OsLOX7, LOX7 | 2.605282485 | 6.085106381 | 1.37E-04 | 0.017224 | 4h | Up |
| Os08g0509100 | Similar to Lipoxygenase, chloroplast precursor (EC 1.13.11.12). (Os08t0509100-01);Similar to Lipoxygenase. (Os08t0509100-02) | OsLOX8, OsLOX9, LOX9, OsLOX2;3, LOX2;3, OsLOX2, OsLOX1, CM-LOX8 | 2.601047205 | 6.0672687 | 2.02E-05 | 0.004627 | 4h | Up |
| Os03g0180900 | Jasmonate ZIM-domain containing protein, Transcriptional repressor of JA signaling, Regulation of phosphate starvation responses, Pi homeostasis (Os03t0180900-01) | OsJAZ2, JAZ2, OsJAZ 11, OsJAZ11, Os-JAZ11, JAZ11, OsTIFY11c, OsJAZ1, JAZ1 | 2.562751114 | 5.908332892 | 1.43E-08 | 2.12E-05 | 4h | Up |
| Os11g0679700 | Glycerol-3-phosphate acyltransferase, Anther development, Pollen formation (Os11t0679700-01) | OsGPAT3 | 2.554903792 | 5.876282653 | 9.10E-06 | 0.002593 | 4h | Up |
| Os01g0609300 | PDR-type ABC transporter 9 (Os01t0609300-01);Hypothetical conserved gene. (Os01t0609300-02) | OsPDR9, ospdr9, OsPDR3, PDR3, OsABCG36/OsPDR9, OsABCG36 | 2.554483774 | 5.874572115 | 3.09E-05 | 0.006057 | 4h | Up |
| Os06g0649000 | PAMP (pathogen-associated molecular pattern)-responsive transrepressor, Defense response (Os06t0649000-01) | OsWRKY28, DLN170, OsDLN170 | 2.462440076 | 5.511481136 | 6.45E-06 | 0.002034 | 4h | Up |
| Os01g0816100 | Plant-specific transcription factor, Regulation of hypersensitive response (HR) cell death (Os01t0816100-01) | OsNAC4, NAC68, ONAC068, OnNAC4*, OnNAC4, OsNAC4/ONAC068 | 2.429343195 | 5.386481488 | 5.27E-07 | 3.56E-04 | 4h | Up |
| Os01g0959100 | Similar to Abscisic stress ripening protein 1. (Os01t0959100-01) | Asr3, OsASR5, OsASR1, Asr1 | 2.366184369 | 5.15575736 | 4.39E-05 | 0.007683 | 4h | Up |
| Os07g0127500 | Similar to PR-1a pathogenesis related protein (Hv-1a) precursor. (Os07t0127500-01) | OsPR1#072, OsPR1-72, PR1L, OsPR1L, OsPRB1, PRB1 | 2.315443291 | 4.977575819 | 5.51E-04 | 0.049439 | 4h | Up |
| Os04g0179100 | Similar to OSIGBa0109M01.2 protein. (Os04t0179100-01) | OsSDR110C-MS2, OsMAS1, MAS1, OsMS2, MS2, MAS2 | 2.300048475 | 4.924743124 | 4.80E-05 | 0.008166 | 4h | Up |
| Os10g0528300 | Tau class glutathione S-transferase 4 (Os10t0528300-01) | OsGSTU4, GST4 | 2.299782358 | 4.923834796 | 4.33E-04 | 0.042817 | 4h | Up |
| Os05g0370700 | Alpha/beta hydrolase family protein. (Os05t0370700-01) |  | 2.264559395 | 4.805076484 | 9.01E-06 | 0.002593 | 4h | Up |
| Os09g0341600 | Cyclin-like F-box domain containing protein. (Os09t0341600-01) | OsFbox478, Os_F0354, OsFBX319, FBX319 | 2.262633725 | 4.798667078 | 3.59E-08 | 4.47E-05 | 4h | Up |
| Os07g0496250 | Similar to Expansin-like B1. (Os07t0496250-01) | Os-EXPR1, OsEXPR1 | 2.253402881 | 4.768061621 | 2.74E-04 | 0.030278 | 4h | Up |
| Os01g0597800 | UDP-glucuronosyl/UDP-glucosyltransferase family protein. (Os01t0597800-01) |  | 2.164385787 | 4.482755416 | 2.23E-05 | 0.004929 | 4h | Up |
| Os04g0179200 | Similar to Stem secoisolariciresinol dehydrogenase (Fragment). (Os04t0179200-01) | OsMAS, MAS1, OsMAS1, OsMAS/SDR110C-MS1, SDR110C-MS1, OsSDR110C-MS1, OsMS1, MS1 | 2.140347779 | 4.408683102 | 2.31E-04 | 0.026388 | 4h | Up |
| Os06g0185100 | Similar to estradiol 17-beta-dehydrogenase 8. (Os06t0185100-00) |  | 2.137041529 | 4.398591212 | 4.56E-06 | 0.00161 | 4h | Up |
| Os05g0442400 | R-R-type MYB-like transcription factor, Response to drought stress during reproductive development (Os05t0442400-01) | OsMYB-1, MYB-1, MYB1, OsARM1, Os2R_MYB56, 2R_MYB56, OsMYBxoc1, MYBxoc1, MYB2-66, OsMYB2-66 | 2.115488518 | 4.333367275 | 3.46E-04 | 0.03577 | 4h | Up |
| Os03g0830500 | FW2.2-like (FWL) family protein, Regulation of cadmium and micronutrient metal accumulation (Os03t0830500-01) | OsFWL7, PGPS/D12, PGPS, D12, OsFWL6, FWL6 | 2.11417061 | 4.329410534 | 1.20E-05 | 0.00314 | 4h | Up |
| Os03g0739700 | Uncharacterised protein family UPF0114 domain containing protein. (Os03t0739700-01) |  | 2.099660001 | 4.286083634 | 3.25E-07 | 2.40E-04 | 4h | Up |
| Os09g0444900 | Similar to plant viral-response family protein. (Os09t0444900-01);Similar to plant viral-response family protein. (Os09t0444900-02) |  | 2.031470439 | 4.088213212 | 8.40E-06 | 0.002452 | 4h | Up |
| Os04g0688500 | Peroxidase (EC 1.11.1.7). (Os04t0688500-01) | prx62, OsPRX62 | 2.029540931 | 4.082749154 | 2.05E-05 | 0.004663 | 4h | Up |
| Os08g0482600 | Cd(II) transporter, Cadmium accumulation, Oxidative stress resistance (Os08t0482600-01) | OsUCL29, BCP1, OsBCP1 | 2.028298616 | 4.079234986 | 1.90E-04 | 0.022383 | 4h | Up |
| Os03g0183500 | FCS-like zinc finger (FLZ) protein 10, Submergence response (Os03t0183500-01) | OsFLZ10 | 2.021090471 | 4.058904711 | 4.45E-06 | 0.001595 | 4h | Up |
| Os10g0392400 | Tify domain containing protein. (Os10t0392400-01) | OsTIFY11d, OsJAZ1, JAZ1, OsJAZ 12, OsJAZ12, JAZ12 | 2.018444531 | 4.051467398 | 5.52E-07 | 3.63E-04 | 4h | Up |
| Os03g0225900 | Allene oxide synthase (CYP74A2), Biosynthesis of jasmonic acid (JA) (Os03t0225900-01);Allene oxide synthase. (Os03t0225900-02) | OsAOS2, CYP74A2, OsAOS, OsAOS1 | 1.991218369 | 3.975726101 | 4.32E-05 | 0.007683 | 4h | Up |
| Os12g0150200 | Cytochrome P450 enzyme, Salt tolerance (Os12t0150200-01) | OsCYP94C2b | 1.985277737 | 3.959388786 | 5.81E-05 | 0.009218 | 4h | Up |
| Os01g0699600 | Mitogen-activated protein kinase kinase kinase (MAPKKK), Negative regulation of seed dormancy (Os01t0699600-01) | OsNPK1-PK, NPK1-PK, OsMKKK62, MKKK62, OsMAPKKK62, MAPKKK62, MEK7, OsMEK7 | 1.982098721 | 3.950673774 | 2.58E-07 | 2.10E-04 | 4h | Up |
| Os06g0586000 | Conserved hypothetical protein. (Os06t0586000-01) |  | 1.947014451 | 3.855757853 | 1.07E-04 | 0.014354 | 4h | Up |
| Os02g0561400 | RING-H2 type E3 ubiquitin ligase, Root development, Maintenance of cell viability after the initiation of root primordial formation, Defense response (Os02t0561400-01) | EL5 | 1.946670478 | 3.854838657 | 3.01E-06 | 0.001228 | 4h | Up |
| Os05g0546300 | Hypothetical protein. (Os05t0546300-01) |  | 1.885467372 | 3.694725984 | 4.01E-04 | 0.040197 | 4h | Up |
| Os08g0137900 | Similar to Chemocyanin precursor (Basic blue protein) (Plantacyanin). (Os08t0137900-01) | OsUCL25 | 1.849599216 | 3.604000511 | 7.14E-07 | 4.22E-04 | 4h | Up |
| Os06g0504900 | WRKY transcription factor 31, Bacterial leaf blight resistance (Os06t0504900-01) | OsWRKY31 | 1.846718546 | 3.596811482 | 3.08E-05 | 0.006057 | 4h | Up |
| Os02g0467600 | Similar to Cinnamate 4-hydroxylase CYP73. (Os02t0467600-01) | OsC4H1, OsC4H, C4H | 1.835731174 | 3.569522694 | 1.75E-09 | 4.59E-06 | 4h | Up |
| Os03g0830400 | PGPS/D12. (Os03t0830400-01) | OsFWL, FWL, OsFWL4/OsPCR6, OsFWL4, OsPCR6, PCR6 | 1.82913818 | 3.553247494 | 1.11E-05 | 0.003012 | 4h | Up |
| Os03g0664800 | Acyl-CoA N-acyltransferase domain containing protein. (Os03t0664800-01) |  | 1.806369905 | 3.497611149 | 1.99E-06 | 9.06E-04 | 4h | Up |
| Os03g0181100 | TIFY family protein, Jasmonate ZIM-domain (JAZ) protein, miR156-target gene, Signal transduction induced by hydrogen peroxide (Os03t0181100-01);Tify domain containing protein. (Os03t0181100-02) | OsJAZ4, JAZ4, OsJAZ 10, OsJAZ10, JAZ10, OsTIFY11b | 1.803438781 | 3.490512265 | 1.22E-09 | 4.03E-06 | 4h | Up |
| Os09g0255400 | Similar to Indole-3-glycerol phosphate synthase, chloroplast precursor (EC 4.1.1.48) (IGPS). (Os09t0255400-01);Similar to indole-3-glycerol phosphate synthase. (Os09t0255400-02) | OsIGPS, TRPC, OsTRPC | 1.785728844 | 3.447926077 | 3.76E-06 | 0.001458 | 4h | Up |
| Os05g0257100 | LRR-receptor-like kinase (LRR-RLK) family protein (Os05t0257100-01);Similar to SHR5-receptor-like kinase (Fragment). (Os05t0257100-02) | OsMRLK24 | 1.755162224 | 3.375642738 | 7.91E-05 | 0.011661 | 4h | Up |
| Os02g0209300 | Hypothetical conserved gene. (Os02t0209300-01) |  | 1.75219645 | 3.368710497 | 2.18E-08 | 3.03E-05 | 4h | Up |
| Os06g0215500 | Similar to Oxo-phytodienoic acid reductase. (Os06t0215500-01) | OsOPR6, OsOPR4, OsOPR06-1 | 1.714607143 | 3.282072579 | 5.06E-04 | 0.04696 | 4h | Up |
| Os03g0820400 | C2H2-type zinc finger protein, Positive regulation of of ABA catabolism, Salinity and drought tolerance, Seed germination (Os03t0820400-01) | ZFP 15, OsZFP15, OsZOS3-22 | 1.712444747 | 3.277156903 | 2.30E-05 | 0.004996 | 4h | Up |
| Os03g0180800 | TIFY domain-containing transcriptional regulator, Salt and dehydration stress tolerance (Os03t0180800-01) | OsJAZ3, JAZ3, OsJAZ 9, OsJAZ9, Os-JAZ9, JAZ9, OsTIFY11a, OsJAZ6, JAZ6 | 1.711496175 | 3.27500288 | 6.16E-05 | 0.009582 | 4h | Up |
| Os10g0520100 | Cyclin-like F-box domain containing protein. (Os10t0520100-00) | OsFbox559, Os_F0572, OsFBDUF49, FBDUF49 | 1.678894456 | 3.201824997 | 7.47E-08 | 8.03E-05 | 4h | Up |
| Os01g0810600 | Similar to cDNA, clone: J100063K14, full insert sequence. (Os01t0810600-00) | OsRLCK46, OsMRLK5, MRLK5 | 1.678215382 | 3.200318258 | 5.45E-06 | 0.001789 | 4h | Up |
| Os03g0853200 | Tetraspanin 6, Control of rice architecture by regulating plant height and tiller number, Response to pathogens (Os03t0853200-01) | OsTET6 | 1.666295522 | 3.173985467 | 1.66E-06 | 7.85E-04 | 4h | Up |
| Os03g0283200 | Similar to IN2-1 protein. (Os03t0283200-00) | OsGSTL1, GST1 | 1.656978104 | 3.153552835 | 1.22E-05 | 0.003143 | 4h | Up |
| Os05g0583000 | Similar to WRKY8. (Os05t0583000-01) | OsWRKY8 | 1.64794482 | 3.133868883 | 3.28E-04 | 0.034621 | 4h | Up |
| Os01g0557100 | Alpha/beta hydrolase family protein. (Os01t0557100-01) |  | 1.64712075 | 3.13207932 | 1.95E-04 | 0.022661 | 4h | Up |
| Os11g0608300 | Barley stem rust resistance protein. (Os11t0608300-01) | OsRLCK337 | 1.63627048 | 3.10861183 | 8.47E-05 | 0.012287 | 4h | Up |
| Os01g0186000 | WRKY transcription factor 10, Negative regulation of thermotolerance (Os01t0186000-01) | OsWRKY10 | 1.628196152 | 3.091262465 | 1.08E-06 | 5.92E-04 | 4h | Up |
| Os08g0404500 | C1-like domain containing protein. (Os08t0404500-01);C1-like domain containing protein. (Os08t0404500-02) |  | 1.623697188 | 3.08163755 | 7.29E-05 | 0.010912 | 4h | Up |
| Os02g0134200 | DUF1645 family protein, Regulation of stress-tolerance and grain length (Os02t0134200-01) | OsSGL | 1.618254969 | 3.070034715 | 4.05E-08 | 4.79E-05 | 4h | Up |
| Os05g0576600 | Conserved hypothetical protein. (Os05t0576600-01) |  | 1.616496901 | 3.066295848 | 2.50E-07 | 2.10E-04 | 4h | Up |
| Os03g0807900 | Chaperonin-like RbcX family protein. (Os03t0807900-01);Chaperonin-like RbcX family protein. (Os03t0807900-02) |  | 1.60811683 | 3.048536522 | 4.41E-05 | 0.007683 | 4h | Up |
| Os03g0790500 | Carboxylesterase, Response to osmotic and drought stress, Response to ABA treatment (Os03t0790500-01) | GID1, GID1L2, CXE3.3, OsCXE3.3 | 1.598724979 | 3.028755204 | 3.29E-06 | 0.001321 | 4h | Up |
| Os05g0324700 | Conserved hypothetical protein. (Os05t0324700-01) |  | 1.585746872 | 3.001631497 | 4.07E-07 | 2.92E-04 | 4h | Up |
| Os07g0638400 | 1 Cys-peroxiredoxin B, Root development (Os07t0638400-01) | Os1-Cys Prx B, 1-Cys Prx B, Os1-CysPrxB, 1-CysPrxB | 1.550518502 | 2.92922396 | 2.00E-04 | 0.023158 | 4h | Up |
| Os06g0536100 | Hypothetical conserved gene. (Os06t0536100-00) | OsRING196 | 1.544408525 | 2.916844597 | 2.10E-05 | 0.004735 | 4h | Up |
| Os10g0491400 | Conserved hypothetical protein. (Os10t0491400-01) |  | 1.54291862 | 2.913833858 | 1.31E-05 | 0.003334 | 4h | Up |
| Os10g0535800 | Protein of unknown function Cys-rich domain containing protein. (Os10t0535800-01) |  | 1.532150274 | 2.892165826 | 3.86E-06 | 0.001471 | 4h | Up |
| Os09g0555150 | Sulfotransferase family protein. (Os09t0555150-01) |  | 1.530529857 | 2.888919206 | 1.66E-04 | 0.019822 | 4h | Up |
| Os01g0214500 | Conserved hypothetical protein. (Os01t0214500-01) |  | 1.52568744 | 2.879238782 | 4.15E-09 | 8.18E-06 | 4h | Up |
| Os04g0405300 | Similar to Stem secoisolariciresinol dehydrogenase (Fragment). (Os04t0405300-01) | OsSDR110C-MS3 | 1.517794599 | 2.863529765 | 4.76E-04 | 0.045427 | 4h | Up |
| Os10g0517500 | Cystathionine γ-lyase (Os10t0517500-02) | OsRRJ1 | 1.513060092 | 2.854147893 | 3.38E-04 | 0.035351 | 4h | Up |
| Os05g0104200 | Polygalacturonase inhibiting protein, Inhibitor of fungal polygalacturonase, Sheath blight resistance (Os05t0104200-01) | Ospgip1, pgip1, OsPGIP1 | 1.498023384 | 2.824554591 | 8.09E-08 | 8.12E-05 | 4h | Up |
| Os04g0395800 | JAZ (JASMONATE ZIM-DOMAIN) protein 5, Dufulin (immuno-inducer)-binding protein, Salt tolerance (Os04t0395800-01) | OsJAZ11, OsJAZ5, OsTIFY9, JAZ11, JAZ5, JAZ11/TIFY9 | 1.485506736 | 2.800155093 | 1.58E-06 | 7.80E-04 | 4h | Up |
| Os05g0130100 | Hypothetical conserved gene. (Os05t0130100-00) |  | 1.471994051 | 2.774050499 | 5.37E-06 | 0.001787 | 4h | Up |
| Os10g0379100 | Tyramine hydroxycinnamoyl transferase 1, Rice immunity, Biosynthesis of phenolamide (Os10t0379100-01) | Os-THT1, OsTHT1 | 1.467297668 | 2.765034858 | 1.06E-04 | 0.014286 | 4h | Up |
| Os05g0537100 | WRKY transcription factor 7, Response to the rice pathogens (Os05t0537100-01) | OsWRKY7 | 1.456897774 | 2.745174339 | 1.02E-04 | 0.014104 | 4h | Up |
| Os01g0864500 | Putative harpin-induced protein, Negative regulation of salt and drought tolerance (Os01t0864500-01) | OsSDS1 | 1.448631423 | 2.729490022 | 2.23E-06 | 9.77E-04 | 4h | Up |
| Os02g0466400 | Similar to Inositol phosphate kinase. (Os02t0466400-01) | OsITPK4, OsITP5/6K-4, ITP5/6K-4 | 1.43942131 | 2.712120558 | 4.52E-04 | 0.043847 | 4h | Up |
| Os12g0111800 | Similar to Class III peroxidase 136. (Os12t0111800-00) |  | 1.405752638 | 2.649559706 | 3.70E-04 | 0.037669 | 4h | Up |
| Os03g0741100 | Basic helix-loop-helix transcription factor, Drought tolerance (Os03t0741100-01) | OsbHLH148, bHLH148 | 1.366828646 | 2.579030162 | 3.46E-04 | 0.03577 | 4h | Up |
| Os01g0160800 | Ribosome-inactivating protein, Defense against herbivorous insects (Os01t0160800-01) | OsRIP1, RIP1, OsjRIP1.1 | 1.365527571 | 2.576705348 | 3.35E-04 | 0.035167 | 4h | Up |
| Os02g0205500 | Similar to Fatty acid elongase 1. (Os02t0205500-01) | KCS, OsKCS2 | 1.365307094 | 2.576311598 | 3.10E-05 | 0.006057 | 4h | Up |
| Os05g0183100 | WRKY transcription factor, Positive regulation of blast and bacteria blight resistance (Os05t0183100-01) | OsWRKY67 | 1.350050875 | 2.549211148 | 4.23E-06 | 0.001538 | 4h | Up |
| Os02g0682300 | RING-type E3 ligase, Negative regulation of cuticular wax biosynthesis, Drought stress response (Os02t0682300-01) | OsRING46, RING46 | 1.344329396 | 2.539121437 | 2.59E-05 | 0.005425 | 4h | Up |
| Os07g0664400 | Short-chain dehydrogenase/reductase SDR domain containing protein. (Os07t0664400-01) |  | 1.333929534 | 2.520883652 | 5.27E-05 | 0.008649 | 4h | Up |
| Os07g0192000 | ATPase, AAA-type, core domain containing protein. (Os07t0192000-01) |  | 1.325862542 | 2.506827182 | 1.85E-05 | 0.004425 | 4h | Up |
| Os11g0483000 | Cytochrome P450, Oxidase, JA-mediated chilling tolerance (Os11t0483000-01) | OsHAN1, OsCYP94B4, CYP94B4 | 1.313673224 | 2.485736244 | 1.59E-05 | 0.003957 | 4h | Up |
| Os01g0947000 | Similar to Beta-1,3-glucanase precursor. (Os01t0947000-01) |  | 1.312614709 | 2.483913113 | 2.87E-05 | 0.005792 | 4h | Up |
| Os02g0624300 | R2R3-type MYB transcription factor, Negative regulation of cold tolerance, Maintenance of Pi homeostasis (Os02t0624300-01) | OsMYB30, OsMYB5P, MYB5P, OsMYB13a, MYB13a, Os2R_MYB26, 2R_MYB26, OsMYB30C, MYB30C, MYB2-29, OsMYB2-29 | 1.312342366 | 2.483444259 | 3.79E-04 | 0.038251 | 4h | Up |
| Os10g0137300 | Similar to BLN1-1. (Os10t0137300-00) |  | 1.304304671 | 2.469646703 | 5.40E-04 | 0.049056 | 4h | Up |
| Os02g0181300 | WRKY transcription factor, Defense response (Os02t0181300-01) | OsWRKY71, WRKY-71 | 1.275333334 | 2.42054738 | 4.15E-06 | 0.001534 | 4h | Up |
| Os01g0934800 | Alpha/beta hydrolase fold-1 domain containing protein. (Os01t0934800-00) | Pir7b | 1.266367908 | 2.40555187 | 3.79E-05 | 0.007063 | 4h | Up |
| Os08g0155900 | Pathogen-induced defense-responsive protein, Negative regulation of disease resistance (Os08t0155900-01) | OsDR10 | 1.26440901 | 2.402287818 | 2.16E-04 | 0.024874 | 4h | Up |
| Os09g0517200 | Nucleotide-binding LRR receptor (NLR) family protein, Regulation of the symbiosis between rice and leaf folder by balancing plant growth and defense (Os09t0517200-01) |  | 1.263787474 | 2.401253096 | 2.58E-04 | 0.029013 | 4h | Up |
| Os12g0518200 | DUF gene family protein, Dufulin-induced promotion of salt stress tolerance (Os12t0518200-01) | OsFCO9, OsDUF6, DUF6 | 1.250444893 | 2.379147789 | 3.43E-04 | 0.035768 | 4h | Up |
| Os01g0389200 | Protein of unknown function DUF679 family protein. (Os01t0389200-01) |  | 1.242697698 | 2.366406135 | 1.92E-04 | 0.022527 | 4h | Up |
| Os06g0522300 | Haem peroxidase family protein. (Os06t0522300-01) | prx81, OsPRX81 | 1.220279022 | 2.329917743 | 2.76E-05 | 0.005631 | 4h | Up |
| Os06g0239200 | Conserved hypothetical protein. (Os06t0239200-00) |  | 1.21400979 | 2.319815051 | 5.68E-07 | 3.63E-04 | 4h | Up |
| Os01g0627500 | Cytochrome P450 family protein. (Os01t0627500-01) | OsCYP72A18 | 1.193798943 | 2.287543127 | 1.64E-05 | 0.004043 | 4h | Up |
| Os08g0477100 | Patatin-related phospholipase AIIepsilon, Patatin-related phospholipase AIIε (Os08t0477100-01) | OspPLAIIepsilon, pPLAIIepsilon | 1.192460042 | 2.285421146 | 1.53E-04 | 0.0185 | 4h | Up |
| Os03g0339400 | Haem peroxidase, plant/fungal/bacterial family protein. (Os03t0339400-01) | prx40, Os PRX40, POD40, OsPOD40 | 1.186230397 | 2.275573834 | 5.33E-14 | 6.30E-10 | 4h | Up |
| Os02g0654700 | AP2/ERF family protein, Abiotic stress response (Os02t0654700-01) | OsERF#091, OsERF091, OsERF91, AP59, OsAP59, AP2/EREBP#147, AP2/EREBP147, OsBIERF3, BIERF3, OsERF3 | 1.18621846 | 2.275555006 | 1.00E-04 | 0.014002 | 4h | Up |
| Os04g0677300 | Harpin-induced 1 domain containing protein. (Os04t0677300-01) |  | 1.17590779 | 2.259350018 | 3.02E-04 | 0.032601 | 4h | Up |
| Os06g0164900 | S-Domain receptor like kinase-66, Partial S-domain containing protein, Response to chilling and submergence (Os06t0164900-00) | SDRLK-66, OsSDRLK-66, OsSDRLK66 | 1.173380942 | 2.25539628 | 3.63E-05 | 0.006925 | 4h | Up |
| Os06g0215900 | Similar to Oxo-phytodienoic acid reductase. (Os06t0215900-01);Similar to 12-oxophytodienoic acid reductase. (Os06t0215900-02) | OsOPR4, OsOPR10, OsOPR06-3, OsOPR7 | 1.171149891 | 2.251911126 | 1.00E-06 | 5.64E-04 | 4h | Up |
| Os01g0639600 | Protein of unknown function DUF1645 family protein. (Os01t0639600-01) |  | 1.1643372 | 2.241302226 | 2.41E-06 | 0.001022 | 4h | Up |
| Os08g0203201 | Similar to SHR5-receptor-like kinase (Fragment). |  | 1.145835606 | 2.212742552 | 2.55E-05 | 0.005393 | 4h | Up |
| Os01g0369700 | Similar to Glutathione S-transferase GST 8 (EC 2.5.1.18). (Os01t0369700-01);Similar to Glutathione S-transferase GST 8 (EC 2.5.1.18). (Os01t0369700-03) | OsGSTF5, OsGST F5 | 1.145589653 | 2.212365352 | 3.83E-05 | 0.00707 | 4h | Up |
| Os06g0185500 | Transferase family protein. (Os06t0185500-00) | OsHCT4 | 1.144599896 | 2.210848085 | 6.82E-05 | 0.010273 | 4h | Up |
| Os11g0684000 | Myb transcription factor, Jasmonate-dependent defense response, Negative regulation of bacterial blight resistance (Os11t0684000-01) | OsJAmyb, JAmyb, OsJAMyb, OsMYB21, MYB21, Os2R_MYB91, 2R_MYB91, OgSH11, OsSH11, MYB2-109, OsMYB2-109 | 1.1439398 | 2.209836757 | 2.21E-05 | 0.004929 | 4h | Up |
| Os01g0314800 | Late embryogenesis abundant protein 9, Negative regulation of cold tolerance (Os01t0314800-01) | OsLEA9, OsLEA5, LEA5 | 1.142247436 | 2.207246011 | 1.06E-04 | 0.014286 | 4h | Up |
| Os08g0203100 | Hypothetical conserved gene. (Os08t0203100-01);LRR-receptor-like kinase (LRR-RLK) family protein (Os08t0203100-02) | OsMRLK44 | 1.140814831 | 2.205055291 | 7.27E-06 | 0.002178 | 4h | Up |
| Os11g0210201 | Conserved hypothetical protein. (Os11t0210201-00) | RZ53 | 1.135557863 | 2.197035015 | 5.01E-04 | 0.04685 | 4h | Up |
| Os08g0120600 | Similar to Fructose-bisphosphate aldolase, cytoplasmic isozyme (EC 4.1.2.13). (Os08t0120600-01) |  | 1.12521831 | 2.181345524 | 9.80E-05 | 0.013799 | 4h | Up |
| Os07g0175500 | Similar to lipid binding protein. (Os07t0175500-00) | OsLTPG17, OsLTPg17 | 1.123051958 | 2.178072472 | 1.09E-17 | 2.58E-13 | 4h | Up |
| Os06g0216300 | Similar to 12-oxophytodienoic acid reductase. (Os06t0216300-01);12-oxophytodienoic acid reductase 1, Jasmonic acid (JA) biosynthesis, Defense response (Os06t0216300-02) | OsOPR1, OsOPR2, OsOPR11, OsOPR06-6, OPDAR1, OsOPR, OPDAR | 1.095819258 | 2.137344197 | 4.04E-05 | 0.007294 | 4h | Up |
| Os01g0748950 | Nitrate transporter of the NRT1/NPF family, Arbuscular mycorrhizal (AM)-induced nitrate transporter, Mycorrhizal nitrate acquisition, Symbiotic nitrogen uptake (Os01t0748950-01) | PTR, OsNPF4.5 | 1.094729516 | 2.135730361 | 3.65E-05 | 0.006925 | 4h | Up |
| Os01g0955100 | Calmodulin-like protein, Drought and salt tolerance (Os01t0955100-01) | OsCML31, OsMSR2, MSR2 | 1.094668824 | 2.135640515 | 8.46E-05 | 0.012287 | 4h | Up |
| Os10g0538200 | Peptidase aspartic, catalytic domain containing protein. (Os10t0538200-01) |  | 1.092389719 | 2.13226939 | 1.50E-04 | 0.018433 | 4h | Up |
| Os01g0836600 | ATP-binding cassette (ABC) transporter, Half-size ABC transporter of the G family, Pollen wall formation (Os01t0836600-01) | OsABCG3 | 1.087355181 | 2.12484143 | 9.29E-09 | 1.57E-05 | 4h | Up |
| Os04g0469000 | Heavy metal-associated isoprenylated plant protein 19, Putative virulence target, Disease resistance (Os04t0469000-01) | OsHIPP19, OsaHIP19, HIP19 | 1.086748564 | 2.123948175 | 8.37E-06 | 0.002452 | 4h | Up |
| Os06g0666400 | Valine-glutamine (VQ) motif-containing protein, Negative regulation of species-non-specific (SNS) broad-spectrum resistance (BSR) (Os06t0666400-01) | OsVQ25 | 1.054443289 | 2.076916606 | 2.81E-06 | 0.001165 | 4h | Up |
| Os04g0121800 | Hypothetical conserved gene. (Os04t0121800-01) |  | 1.051348461 | 2.072466039 | 4.60E-04 | 0.04418 | 4h | Up |
| Os07g0130800 | Protein kinase, catalytic domain domain containing protein. (Os07t0130800-01) |  | 1.050513172 | 2.071266474 | 1.33E-04 | 0.016824 | 4h | Up |
| Os07g0522500 | ATP binding cassette (ABC)-type transporter, Cadmium tolerance (Os07t0522500-01) | ABCG43, OsABCG43, ABCG43/PDR5, OsPDR5 | 1.050100163 | 2.070673605 | 2.57E-04 | 0.029013 | 4h | Up |
| Os02g0627100 | Phenylalanine ammonia-lyase, Broad spectrum disease resistance (Os02t0627100-01) | OsPAL1, PAL, OsPAL06, ZB8, pal/zb8, PAL06, rPAL-P5, ZB8PAL, OsPAL, OsPAL2, OsPAL4, PAL4, PAL/ZB8, OsPAL6, PAL6, OsPAL8, PAL8 | 1.049677172 | 2.070066583 | 5.64E-05 | 0.009135 | 4h | Up |
| Os01g0548600 | Protein kinase, catalytic domain domain containing protein. (Os01t0548600-01) |  | 1.049475041 | 2.069776573 | 5.76E-05 | 0.009198 | 4h | Up |
| Os03g0571900 | Phenolics efflux transporter, Essential for the utilization of apoplasmic precipitated iron in the stele (Os03t0571900-01) | OsPEZ1, OsMATE14, MATE14 | 1.048111157 | 2.067820788 | 8.73E-05 | 0.012592 | 4h | Up |
| Os01g0713200 | Similar to Beta-glucanase. (Os01t0713200-01) | Gns10, OsGns10 | 1.042930727 | 2.060408972 | 1.15E-04 | 0.015206 | 4h | Up |
| Os03g0576200 | High affinity K+ transporter (HAK), Maintenance of ion homeostasis, Salinity tolerance (Os03t0576200-01) | OsHAK21 | 1.041738342 | 2.05870675 | 6.39E-05 | 0.009813 | 4h | Up |
| Os07g0597200 | LRR-receptor-like kinase (LRR-RLK) family protein (Os07t0597200-01);Protein kinase, core domain containing protein. (Os07t0597200-02) | OsBIN1 | 1.038550127 | 2.054162234 | 1.14E-05 | 0.00305 | 4h | Up |
| Os01g0714600 | Similar to cDNA clone:J023088C01, full insert sequence. (Os01t0714600-01) |  | 1.028876762 | 2.040435012 | 1.19E-04 | 0.015495 | 4h | Up |
| Os02g0740600 | Conserved hypothetical protein. (Os02t0740600-01) |  | 1.028340561 | 2.039676793 | 3.06E-04 | 0.032907 | 4h | Up |
| Os10g0528400 | Tau class glutathione S-transferase 3 (Os10t0528400-01) | OsGSTU3, OsGSTU29, GSTU29 | 1.027156337 | 2.038003229 | 1.49E-04 | 0.018333 | 4h | Up |
| Os07g0162400 | Alpha/beta hydrolase fold-3 domain containing protein. (Os07t0162400-01) | OsCDAP1, GID1L2, CXE7.1, OsCXE7.1 | 1.019425697 | 2.027111853 | 6.53E-05 | 0.009969 | 4h | Up |
| Os08g0231400 | Germin-like protein 8-12, Disease resistance (Os08t0231400-01) | OsGLP8-12, OsCDP8.12, CDP8.12 | 1.018893056 | 2.026363585 | 1.49E-05 | 0.003736 | 4h | Up |
| Os01g0601651 | Plant disease resistance response protein domain containing protein. (Os01t0601651-01) |  | 1.013221702 | 2.018413418 | 4.17E-04 | 0.041474 | 4h | Up |
| Os08g0331000 | Conserved hypothetical protein. (Os08t0331000-01) |  | 0.99481649 | 1.992827023 | 2.87E-04 | 0.031306 | 4h | Up |
| Os04g0651000 | Similar to Peroxidase. (Os04t0651000-01) | prx57 | 0.991037664 | 1.987614076 | 5.58E-04 | 0.049637 | 4h | Up |
| Os10g0365200 | Thioredoxin fold domain containing protein. (Os10t0365200-00) | OsGSTU33 | 0.988768865 | 1.984490787 | 5.46E-05 | 0.008898 | 4h | Up |
| Os08g0538600 | Stress up-regulated Nod 19 family protein. (Os08t0538600-01) |  | 0.979639512 | 1.971972608 | 4.91E-05 | 0.008302 | 4h | Up |
| Os04g0556500 | cis-Zeatin-O-glucosyltransferase, Catalyze O-glucosylation of cZ and cZ-riboside (Os04t0556500-01) | OscZOGT1, cZOGT1 | 0.971887438 | 1.961404969 | 4.70E-06 | 0.001635 | 4h | Up |
| Os01g0850900 | SOUL haem-binding protein domain containing protein. (Os01t0850900-01) |  | 0.970200852 | 1.959113324 | 5.32E-04 | 0.048573 | 4h | Up |
| Os01g0871600 | Proton-dependent oligopeptide transporter (POT) family protein, Iron homeostasis, Iron uptake (Os01t0871600-01) | OsPOT, POT, OsIROPT1, IROPT1 | 0.965859289 | 1.953226544 | 1.43E-04 | 0.017879 | 4h | Up |
| Os01g0834900 | Hypothetical conserved gene. (Os01t0834900-01) |  | 0.965391933 | 1.952593905 | 5.21E-05 | 0.00861 | 4h | Up |
| Os05g0472400 | Influx transporter for Zn and Cd, Zinc/cadmium uptake (Os05t0472400-01) | OsZIP9, ZIP9 | 0.964819911 | 1.951819864 | 5.72E-06 | 0.001854 | 4h | Up |
| Os06g0521500 | Haem peroxidase family protein. (Os06t0521500-01);Haem peroxidase family protein. (Os06t0521500-02) | prx83 | 0.961131454 | 1.94683613 | 3.66E-05 | 0.006925 | 4h | Up |
| Os10g0537800 | Aspartic protease, Defense response against fungal, bacterial and viral infections (Os10t0537800-01) | OsAP77 | 0.958698485 | 1.943555742 | 1.07E-04 | 0.014354 | 4h | Up |
| Os04g0447700 | Similar to Polyketide reductase. (Os04t0447700-01) | OsAKR5, AKR5 | 0.957808468 | 1.942357107 | 2.65E-04 | 0.029554 | 4h | Up |
| Os03g0439700 | Protein of unknown function DUF1230 family protein. (Os03t0439700-01) |  | 0.955598457 | 1.939383959 | 4.42E-05 | 0.007683 | 4h | Up |
| Os06g0215600 | Similar to Oxo-phytodienoic acid reductase. (Os06t0215600-02) | OsOPR5, OsOPR6, OsOPR06-2, OPR4 | 0.946779701 | 1.927565254 | 8.85E-05 | 0.012689 | 4h | Up |
| Os09g0412300 | Similar to Calmodulin-like protein. (Os09t0412300-01) |  | 0.939815455 | 1.918282843 | 9.43E-05 | 0.01335 | 4h | Up |
| Os02g0681200 | Zinc finger, RING/FYVE/PHD-type domain containing protein. (Os02t0681200-01) | OsRING327, RING327 | 0.925856259 | 1.899811471 | 1.73E-05 | 0.004213 | 4h | Up |
| Os04g0406600 | Prephenate dehydratase domain containing protein. (Os04t0406600-01) | OsADT, ADT | 0.922083684 | 1.89485005 | 2.97E-05 | 0.005958 | 4h | Up |
| Os11g0703100 | Thaumatin, pathogenesis-related family protein. (Os11t0703100-01) |  | 0.913943333 | 1.884188538 | 4.35E-04 | 0.042827 | 4h | Up |
| Os02g0584700 | Heavy metal transport/detoxification protein domain containing protein. (Os02t0584700-01) | OsHPP3, OsaHPP03, HPP03 | 0.904445838 | 1.871825359 | 2.06E-06 | 9.20E-04 | 4h | Up |
| Os02g0705400 | Similar to Pathogen induced protein 2-4. (Os02t0705400-01);Similar to Pathogen induced protein 2-4. (Os02t0705400-02) | OsUSP11 | 0.896706747 | 1.861811156 | 1.95E-05 | 0.004564 | 4h | Up |
| Os02g0575000 | Conserved hypothetical protein. (Os02t0575000-01) |  | 0.895616728 | 1.860405008 | 4.32E-05 | 0.007683 | 4h | Up |
| Os03g0388600 | R2R3-MYB transcription factor 38 (Os03t0388600-01) | OsMYB5, MYB5, Os2R_MYB38, MYB2-43, OsMYB2-43 | 0.88995598 | 1.85311958 | 1.80E-04 | 0.021283 | 4h | Up |
| Os06g0718400 | Hypothetical conserved gene. (Os06t0718400-00) | OsUCL18 | 0.889715675 | 1.852810937 | 6.60E-06 | 0.002053 | 4h | Up |
| Os08g0448000 | 4-coumarate:coenzyme A ligase, Lignin biosynthesis, Defense against wounding (Os08t0448000-01) | Os4CL5, 4CL3, Os4CL3 | 0.882533869 | 1.843610473 | 4.92E-04 | 0.046585 | 4h | Up |
| Os02g0605900 | Similar to Chitinase (EC 3.2.1.14) A. (Os02t0605900-01) | Cht6 | 0.878601975 | 1.838592773 | 5.52E-04 | 0.049439 | 4h | Up |
| Os06g0146800 | Conserved hypothetical protein. (Os06t0146800-01) |  | 0.861397381 | 1.816797192 | 2.99E-04 | 0.032445 | 4h | Up |
| Os07g0174900 | Plant lipid transfer protein and hydrophobic protein, helical domain containing protein. (Os07t0174900-00) | OsLTPG16, OsLTPg16 | 0.860055191 | 1.815107747 | 3.23E-04 | 0.034226 | 4h | Up |
| Os08g0389700 | Conserved hypothetical protein. (Os08t0389700-01);Protein of unknown function DUF81 family protein. (Os08t0389700-02);Similar to cDNA clone:001-103-F02, full insert sequence. (Os08t0389700-03) |  | 0.859599806 | 1.814534901 | 3.23E-09 | 6.94E-06 | 4h | Up |
| Os06g0721800 | Cupredoxin domain containing protein. (Os06t0721800-01) | OsUCL19 | 0.843275886 | 1.794119375 | 1.11E-04 | 0.014758 | 4h | Up |
| Os07g0142500 | Conserved hypothetical protein. (Os07t0142500-00) |  | 0.818145887 | 1.763138597 | 1.24E-06 | 6.53E-04 | 4h | Up |
| Os01g0106400 | Isoflavone reductase-like protein, Homeostasis of reactive oxygen species (ROS) (Os01t0106400-01) | OsIRL | 0.810704028 | 1.75406721 | 5.41E-04 | 0.049056 | 4h | Up |
| Os05g0161800 | Calcium-activated (p)ppGpp synthetase, Ca2+-activated RelA-SpoT homolog 3 (Os05t0161800-01) | OsCRSH3 | 0.809895851 | 1.753084882 | 3.56E-06 | 0.001402 | 4h | Up |
| Os10g0530900 | Similar to Glutathione S-transferase GST 30 (EC 2.5.1.18). (Os10t0530900-01);Similar to cDNA clone:006-203-F02, full insert sequence. (Os10t0530900-03) | OsGSTU50 | 0.809161956 | 1.752193319 | 4.42E-04 | 0.043399 | 4h | Up |
| Os03g0764100 | C2H2-type zinc-finger protein, Drought stress tolerance (Os03t0764100-01) | WZF1, OsWZF1, DLN99, OsDLN99, OsZFP1, ZFP1, OsZOS3-18 | 0.806208813 | 1.748610314 | 1.28E-04 | 0.016221 | 4h | Up |
| Os04g0182200 | 2OG-Fe(II) oxygenase domain containing protein. (Os04t0182200-01) | 2-ODD23, Os2-ODD23, Os2ODD23 | 0.799978292 | 1.741074929 | 5.58E-04 | 0.049637 | 4h | Up |
| Os03g0709000 | Membrane-associated, eicosanoid and glutathione metabolism (MAPEG) domain containing protein. (Os03t0709000-01) |  | 0.784633116 | 1.722654184 | 6.13E-07 | 3.81E-04 | 4h | Up |
| Os02g0799600 | Conserved hypothetical protein. (Os02t0799600-01) |  | 0.770888782 | 1.70632065 | 5.30E-04 | 0.048547 | 4h | Up |
| Os01g0949750 | Similar to Glutathione S-transferase GST 28 (Fragment). (Os01t0949750-00) | OsGSTU35 | 0.763266132 | 1.697328878 | 4.03E-04 | 0.040197 | 4h | Up |
| Os09g0468300 | Hypothetical conserved gene. (Os09t0468300-00) | OsATL101, OsRING304, RING304 | 0.762404036 | 1.696314927 | 3.80E-04 | 0.038251 | 4h | Up |
| Os03g0850700 | Phosphatidylinositol transfer protein, Class III SEC14 protein (Os03t0850700-01);Similar to Phosphatidylinositol phosphatidylcholine transfer protein sec14 cytosolic-like protein. (Os03t0850700-02) | OsSec14-12, Sec14-12 | 0.738528975 | 1.668473734 | 5.20E-04 | 0.047822 | 4h | Up |
| Os10g0109900 | Major facilitator superfamily, general substrate transporter domain containing protein. (Os10t0109900-00) | PTR | 0.733192228 | 1.662313199 | 1.74E-04 | 0.020706 | 4h | Up |
| Os04g0356600 | S-Domain receptor like kinase-25, Response to Xanthomonas oryzae pv. oryzae. (Os04t0356600-01) | SDRLK-25, OsSDRLK-25, OsSDRLK25 | 0.730521858 | 1.65923917 | 1.01E-04 | 0.014056 | 4h | Up |
| Os01g0655500 | Protein kinase, core domain containing protein. (Os01t0655500-01);Similar to protein kinase domain containing protein. (Os01t0655500-02) |  | 0.727776393 | 1.656084622 | 1.47E-07 | 1.34E-04 | 4h | Up |
| Os03g0793900 | Conserved hypothetical protein. (Os03t0793900-00) | OsLTPG8, OsLTPg8 | 0.723529289 | 1.651216496 | 1.40E-06 | 7.20E-04 | 4h | Up |
| Os10g0580400 | High-affinity urea transporter, Effective urea acquisition and utilisation, Effective use of low external urea as a N source (Os10t0580400-01) | OsDUR3 | 0.72323522 | 1.650879958 | 4.58E-04 | 0.04418 | 4h | Up |
| Os01g0933900 | Similar to Glutathione transferase III(B) (EC 2.5.1.18). (Os01t0933900-01);Similar to Glutathione transferase III(B) (EC 2.5.1.18). (Os01t0933900-02) | OsGSTF4 | 0.721752921 | 1.64918463 | 5.68E-05 | 0.009144 | 4h | Up |
| Os03g0673400 | Conserved hypothetical protein. (Os03t0673400-01) |  | 0.718595574 | 1.645579326 | 4.96E-04 | 0.046688 | 4h | Up |
| Os03g0270000 | AT-hook motif nuclear-localized (AHL) gene family protein 7, Response to drought and salt stress (Os03t0270000-01) | OsAHL7 | 0.696300299 | 1.620344189 | 6.42E-07 | 3.89E-04 | 4h | Up |
| Os07g0516300 | A member of S40 gene family, Leaf senescence, Response to pathogen infection (Os07t0516300-01) | OsS40-15 | 0.685670743 | 1.608449614 | 5.09E-04 | 0.04704 | 4h | Up |
| Os05g0534800 | Hypothetical conserved gene. (Os05t0534800-01) |  | 0.678520783 | 1.600497899 | 1.48E-06 | 7.44E-04 | 4h | Up |
| Os06g0495800 | Protein of unknown function DUF617, plant family protein. (Os06t0495800-01) |  | 0.675480147 | 1.597128229 | 6.43E-06 | 0.002034 | 4h | Up |
| Os10g0576900 | NAD(P)-binding domain containing protein. (Os10t0576900-01) | OsCCR3 | 0.64567144 | 1.56446723 | 6.27E-05 | 0.009698 | 4h | Up |
| Os11g0116300 | Class IV chalcone isomerase (CHI) protein family member, Chalcone isomerase like protein, Biosynthesis of extractable flavones and tricin-lignin (Os11t0116300-01) | CHI, OsCHI6, OsCHIL1, CHIL1 | 0.643412062 | 1.562019064 | 5.47E-04 | 0.049406 | 4h | Up |
| Os03g0793800 | Plant lipid transfer protein and hydrophobic protein, helical domain containing protein. (Os03t0793800-01) | OsLTPG7, OsLTPg7 | 0.632580079 | 1.550335096 | 3.18E-09 | 6.94E-06 | 4h | Up |
| Os02g0173200 | VQ domain containing protein. (Os02t0173200-01) | OsVQ5 | 0.631691689 | 1.549380716 | 1.53E-04 | 0.0185 | 4h | Up |
| Os03g0794000 | Plant lipid transfer protein/Par allergen family protein. (Os03t0794000-01) | OsLTPG9, OsLTPg9 | 0.626842541 | 1.544181721 | 3.57E-04 | 0.03658 | 4h | Up |
| Os08g0114300 | D-arabinono-1,4-lactone oxidase domain containing protein. (Os08t0114300-01) |  | 0.6251776 | 1.542400688 | 2.76E-04 | 0.030333 | 4h | Up |
| Os04g0483500 | Beta-ketoacyl-CoA reductase, Cuticular wax biosynthesis, Fatty acid elongation (Os04t0483500-01) | OsKCR1, OsWSL3, WSL3 | 0.615278242 | 1.531853406 | 6.78E-10 | 2.75E-06 | 4h | Up |
| Os12g0175400 | R2R3-MYB transcription factor 94 (Os12t0175400-01) | R2R3-MYB, OsDLN254, OsMYB2, Os2R_MYB94, 2R_MYB94, MYB2-113, OsMYB2-113 | 0.613512894 | 1.529980106 | 4.80E-06 | 0.001645 | 4h | Up |
| Os02g0646200 | Zinc finger, B-box domain containing protein. (Os02t0646200-01) | OsBBX6 | 0.609172293 | 1.52538381 | 6.62E-05 | 0.010042 | 4h | Up |
| Os03g0194600 | Protein of unknown function DUF568, DOMON-like domain containing protein. (Os03t0194600-01) | OsDUF568.2 | 0.606990057 | 1.523078243 | 3.77E-05 | 0.007063 | 4h | Up |
| Os03g0751100 | Iron-deficiency-regulated oligopeptide transporter, Iron homeostasis (Os03t0751100-01);Similar to Glutathione transporter. (Os03t0751100-02) | OPT, OsOPT7 | 0.605517213 | 1.521524129 | 2.25E-05 | 0.004932 | 4h | Up |
| Os01g0210600 | Protein of unknown function DUF538 family protein. (Os01t0210600-02) |  | 0.595900708 | 1.511415903 | 2.75E-07 | 2.14E-04 | 4h | Up |
| Os02g0706900 | Alpha/beta hydrolase family protein. (Os02t0706900-00) |  | 0.593768427 | 1.509183704 | 2.68E-05 | 0.005536 | 4h | Up |
| Os05g0280500 | Phospholipid/glycerol acyltransferase domain containing protein. (Os05t0280500-01) | GPAT | 0.589857861 | 1.505098453 | 4.50E-04 | 0.043812 | 4h | Up |
| Os01g0126100 | Multicopper oxidase (Os01t0126100-01) | OsLPR1 | -0.761702951 | -1.695490793 | 2.61E-04 | 0.029201 | 4h | Down |
| Os01g0368900 | Glutaredoxin (Grx) family protein, Arsenic (As) stress response, Drought tolerance (Os01t0368900-01) | OsGRX4, OsGrx_C7, Grx_C7 | -0.914477622 | -1.884886459 | 4.86E-04 | 0.046143 | 4h | Down |
| Os01g0795100 | Similar to Subtilase. (Os01t0795100-00) | OsSub7 | -1.288845011 | -2.443323702 | 9.04E-05 | 0.012878 | 4h | Down |
| Os02g0165200 | Hypothetical protein. (Os02t0165200-01) |  | -0.686645438 | -1.609536661 | 3.89E-05 | 0.00713 | 4h | Down |
| Os03g0625300 | Similar to nucleotide binding protein. (Os03t0625300-00) | OsWD40-77 | -1.314594615 | -2.487324292 | 2.79E-04 | 0.030502 | 4h | Down |
| Os04g0686800 | Vacuolar iron transporter homologue, Iron homeostasis (Os04t0686800-01) | OsVTL5, VTL5, OsNL2 | -0.906761334 | -1.874832013 | 1.10E-07 | 1.04E-04 | 4h | Down |
| Os05g0390800 | Similar to VQ motif family protein. (Os05t0390800-00) | OsVQ19 | -0.940558727 | -1.919271391 | 1.24E-04 | 0.015789 | 4h | Down |
